# Gene expression profiling during hibernation in the European hamster

**DOI:** 10.1038/s41598-018-31506-2

**Published:** 2018-09-03

**Authors:** Célia Gautier, Béatrice Bothorel, Dominique Ciocca, Damien Valour, Albane Gaudeau, Clémence Dupré, Giulia Lizzo, Chantal Brasseur, Isabelle Riest-Fery, Jean-Philippe Stephan, Olivier Nosjean, Jean A. Boutin, Sophie-Pénélope Guénin, Valérie Simonneaux

**Affiliations:** 1 PEX Biotechnologie Chimie & Biologie, Institut de Recherches Servier, Croissy sur Seine, France; 2 Institut des Neurosciences Cellulaires et Intégratives, Strasbourg, France; 3 PEX Méthodologie et Valorisation des Données, Institut de Recherches Internationales Servier, Suresnes, France; 4 Institut de Recherches Internationales Servier, Suresnes, France

## Abstract

Hibernation is an exceptional physiological response to a hostile environment, characterized by a seasonal period of torpor cycles involving dramatic reductions of body temperature and metabolism, and arousal back to normothermia. As the mechanisms regulating hibernation are still poorly understood, here we analysed the expression of genes involved in energy homeostasis, torpor regulation, and daily or seasonal timing using digital droplet PCR in various central and peripheral tissues sampled at different stages of torpor/arousal cycles in the European hamster. During torpor, the hypothalamus exhibited strongly down-regulated gene expression, suggesting that hypothalamic functions were reduced during this period of low metabolic activity. During both torpor and arousal, many structures (notably the brown adipose tissue) exhibited altered expression of deiodinases, potentially leading to reduced tissular triiodothyronine availability. During the arousal phase, all analysed tissues showed increased expression of the core clock genes *Per1* and *Per2*. Overall, our data indicated that the hypothalamus and brown adipose tissue were the tissues most affected during the torpor/arousal cycle, and that clock genes may play critical roles in resetting the body’s clocks at the beginning of the active period.

## Introduction

Animals living in the wild must cope with seasonal variations in ambient temperature (Ta) and day length (photoperiod), which impact food availability. These seasonal variations necessitate major physiological adaptations, particularly for endotherms that have to maintain a relatively constant body temperature (Tb). As winter approaches, animals adapt to the decreasing Ta, photoperiod, and food resources by limiting their energy expenditure in various ways, including decreasing their metabolism, inhibiting reproduction, and insulating their body. The most extreme and efficient strategy is hibernation, an adaptive event observed in mammalian groups, including monotremes, bats, primates, and rodents^1,2^. Hibernation reportedly gives these animals a better chance of survival^2^.

Hibernation is an exceptional physiological phenomenon comprising multiple phases within a two-switch model^1,3,4^. The summer-winter switch enables phases of controlled heterothermy that are required to enter a state of torpor. The second switch occurs within the winter period, and controls a series of hypothermia periods—torpor bouts, lasting from a few hours to several days depending on the species—that are interrupted by short spontaneous interbouts of normothermia. Torpor bouts are characterized by low Tb and drastically reduced metabolic activity, with diminution of heartbeat and respiration, altered fuel utilization, and reduction of costly cell processes. Arousal phases involve rapid rewarming and increased metabolic activity. During the normothermic interbouts, Tb and metabolic activity return to a basal level, possibly to clear metabolic waste^5^. Hibernators are divided into two categories based on their energy saving strategy: fat-storing species (e.g. the marmot, *Marmota marmota*) exhibit an extensive fattening period prior to hibernation, while food-storing species (e.g. the European hamster, *Cricetus cricetus*) hoard food in a burrow and feed between torpor bouts. Although these strategies require different metabolic adaptations, they both involve fasting states that last from a few hours to several days or months^6–8^.

In many species, the time of hibernation is controlled by seasonal changes in photoperiod and temperature^9^. Photoperiodic cycles influence the levels of melatonin, a neurohormone that is secreted by the pineal gland only during the night, with correspondingly greater production during long winter nights^10^. Photoperiod-related changes in circulating melatonin are fundamental for seasonal functions, including hibernation^11^. Intracerebroventricular infusion of melatonin prolongs hibernation bouts in golden-mantled ground squirrels *(Citellus lateralis)*^12^ and treatment with the melatonin antagonist S22153 reduces total hibernation duration in Syrian hamster (*Cricetus auratus*)^13^. Studies in mammals have characterized two melatonin receptors with high-affinity binding, MT_1_ and MT_2_, and one with low-affinity binding, *MT*_*3*_ (identified as quinone reductase 2; QR2)^14^. Additionally, GPR50—an orphan receptor derived from Mel1c in amphibians and birds^15,16^—is reportedly involved in torpor in mice^17^.

MT_1_ is present at the highest density in the *pars tuberalis*, a region of the pituitary stalk where melatonin regulates the expression of thyroid-stimulating hormone (TSH) by specific thyrotrope cells^18^. TSH secreted during a long photoperiod reaches TSH receptors expressed by the tanycytes, specialized glial cells lining the wall of the basal part of the third ventricle and sending processes in the mediobasal hypothalamus and median eminence. There, TSH increases deiodinase 2 (Dio2) and inhibits Dio3 expression within the tanycytes, resulting in increased local levels of active triiodothyronine (T3)^19–25^. It is believed that melatonin effects on seasonal functions are enacted through seasonal changes in hypothalamic T3^11,26^. This likely includes hibernation, since central administration of T3 reduces daily torpor in the Siberian hamster (*Phodopus sungorus*)^27^. Rhythmic melatonin expression and other biological rhythms depend on a circadian system comprising a master clock located in the suprachiasmatic nucleus (SCN), and secondary clocks within other central and peripheral structures^28^. Circadian rhythms are generated by transcriptional/translational feedback loops involving core clock genes, such as *Bmal1*, *Clock*, *Cry*, *Per*, and *Rev-Erbα*^29^. While the roles of circadian clocks during hibernation are unknown, during the torpor bout in hibernating European hamsters, clock genes (including *Per1, Per2*, and *Bmal1*) stop ticking and melatonin remains at a constant low level^30^.

During prolonged fasting in hibernating animals, energy is supplied almost exclusively by lipid oxidation, and this switch in fuel utilization from carbohydrates to lipids must be highly regulated^31^. During hibernation, the primary source of endogenous glucose source becomes hepatic gluconeogenesis using lactate, pyruvate, glycerol, and amino acids^32^. In white adipose tissue, triglycerides are catabolized into glycerol and fatty acids, which are released into circulation^33^. Glycerol is converted into glucose, while fatty acids are metabolized into β-hydroxybutyrate and acetoacetate. These ketone bodies and glucose are the only fuel substrates for the brain^34^. This process also involves upregulation of silent information regulator 1 (SIRT1), which reduces adipogenesis by inhibiting the important adipogenic transcription factor peroxisome proliferator-activated receptor gamma (PPAR-γ), and stimulates hepatic gluconeogenesis by promoting deacetylation of PPARγ coactivator-1 alpha (PGC-1α)^35,36^. Peroxisome proliferator-activated receptor alpha (PPARα) also influences energy store management by stimulating hepatic fatty acid oxidation^37^. PPARα regulates the liver-derived fibroblast growth factor 21 (FGF21), which plays a major role in adaptive starvation responses, stimulating lipolysis in white adipose tissue and ketogenesis in liver tissue, and promoting torpor^38,39^. Finally, previous studies indicate that thioredoxin-interacting protein (TXNIP)—a negative regulator of thioredoxin that is involved in hypothalamic homeostasis and hepatic gluconeogenesis^40,41^—is up-regulated in the hypothalamus during torpor in GPR50 KO mice^42^, Siberian hamsters^42^, and ground squirrels^43^.

Another essential part of hibernation is the arousal from torpor bouts, in which brown adipose tissue (BAT) plays a major role by increasing Tb through non-shivering thermogenesis. Thermogenesis in BAT occurs due to the mitochondrial uncoupling protein 1 (UCP1), which uncouples the mitochondrial respiratory chain by catalysing the regulated re-entry of protons into the matrix, leading to reduced ATP synthesis and generation of heat^44^. Indeed, in the thirteen-lined ground squirrel (*Ictidomys tridecemlineatus*), UCP1 is upregulated to a similar degree during both torpor and arousal compared to in the summer active state^45,46^.

Although studies suggest general reductions of cellular metabolism in torpid states, the cellular and molecular pathways that drive the successive drops and rises in metabolic activity remain poorly understood. One approach to elucidating these mechanisms is to search for genes that display differential expressing patterns according to hibernation state. This approach implies that hibernators have no specific set of genes dedicated to hibernation, but rather rely on differential expression of genes that exist in most mammals^47^. Such gene profiling could be performed using an unbiased wide transcriptomic analysis, such RNASeq^43,45,48^, or a candidate genes approach^49^. In our present study, we aimed to establish a molecular signature of hibernation by analysing the expression of genes with potential involvement in energy homeostasis, torpor regulation, and daily or seasonal timing, in eight central and peripheral organs sampled during three different hibernation phases in the European hamster.

## Results

**Figure 1:**
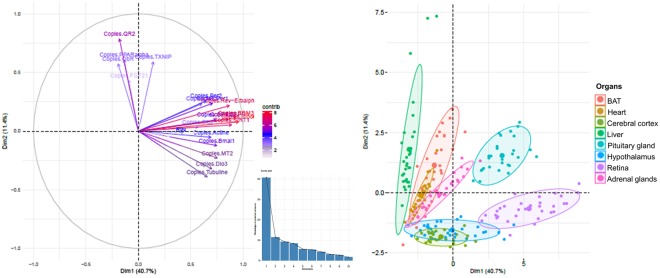
Major sources of variance among the organs, the genes and the hibernation stages of European hamster. The major sources of variance among all the paramaeters were determined by principal component analysis (PCA) of the ddPCR data, are associated with the organ effect. All groups were separated in the space drawn by the two first components (Dim1&2, explaining respectively 40.7% and 11.4% of variance). The variable contribution to the variance is represented with a colour gradient (contrib).

Eight central and peripheral organs (cerebral cortex, hypothalamus, pituitary gland, retina, liver, heart, brown adipose tissue, and adrenal glands) were sampled at three different hibernation phases (normothermia, torpor, and arousal) in male European hamsters. The ddPCR results provided the absolute count of RNA copies encoding melatonin receptors, thyroid metabolism enzymes, clock proteins, and selected proteins involved in energy homeostasis (a summary of the median, quartile and interquartile of absolute RNA copies of each gene in each organ at each hibernation state is given in Table S1). The principal component analysis (PCA) of these ddPCR data revealed that the major sources of variance were associated with the organ effect which groups were separated in the space drawn by the two first components (Dim1&2), explaining respectively 40.7% and 11.4% of variance; as shown in the Fig. 1. Then, Kruskal-Wallis analysis was performed on genes expressed for each organ during the different hibernation phases, revealing 61 significant adjusted *P* value (Benjamini-Hochberg) corresponding to 18 genes according to the hibernation state (Table 1). Thus, the median ratio between all hibernation phases (torpor versus normothermia, arousal versus normothermia and arousal versus torpor) was calculated for each genes and organs in order to more easily visualize gene expression variation (Table 2). All categories of genes and all organs displayed genomic modifications associated with the hibernation states although with some differences between groups.

**Table 1:** List of the 61-significant adjusted *P* value (Benjamini-Hochberg) corresponding to 18 genes according to the hibernation state (Kruskal-Wallis, adjusted *P*-values ≤ 0,05) in a tissue-by-tissue analysis.

Organs	Genes	Adjusted *P* values	Organs	Genes	Adjusted *P* values
Liver	MT2	0.006	Heart	Tubulin	0.019
Liver	ObR	0.006	Pituitary gland	Dio2	0.025
Liver	Tubulin	0.006	Pituitary gland	Per2	0.025
BAT	Dio2	0.008	Pituitary gland	QR2	0.025
BAT	Per2	0.008	Retina	Bmal1	0.028
Retina	FGF21	0.008	Retina	MT2	0.028
Retina	Per2	0.009	Retina	Per1	0.028
Retina	PPARα	0.009	Retina	QR2	0.028
Hypothalamus	PGC1α	0.01	Retina	Tubulin	0.028
Hypothalamus	PPARα	0.01	Hypothalamus	Per1	0.031
Hypothalamus	Tubulin	0.01	Pituitary gland	Bmal1	0.032
Hypothalamus	Actin	0.011	Pituitary gland	Tubulin	0.032
Hypothalamus	G6PD	0.011	Adrenal glands	Per1	0.036
Hypothalamus	Per2	0.011	Cortex	Per1	0.037
Hypothalamus	Bmal1	0.011	BAT	Per1	0.038
Hypothalamus	RBM3	0.011	Hypothalamus	TXNIP	0.038
Hypothalamus	Rev-Erbα	0.011	Heart	Rev-Erbα	0.039
Hypothalamus	MT2	0.012	Pituitary gland	Rev-Erbα	0.041
BAT	PPARα	0.013	Pituitary gland	Clock	0.041
Adrenal glands	FGF21	0.013	Pituitary gland	FGF21	0.041
Adrenal glands	Per2	0.013	Pituitary gland	TXNIP	0.041
Cortex	Per2	0.014	Liver	Per2	0.041
BAT	Dio3	0.014	Heart	Actin	0.042
Hypothalamus	Clock	0.015	Heart	Dio2	0.042
Hypothalamus	QR2	0.015	Heart	TXNIP	0.042
Hypothalamus	SIRT1	0.015	Hypothalamus	FGF21	0.042
BAT	MT2	0.016	BAT	SIRT1	0.045
Liver	Per1	0.018	Liver	FGF21	0.048
Heart	Per1	0.019	Cortex	Dio2	0.049
Heart	Per2	0.020			

Organs/genes combinations are ranked by adjusted *P* -values.

**Table 2:** The median ratio between all groups (Torpor versus Normothermia, Arousal versus Normothermia and Arousal versus Torpor) was calculated for each genes and organs (N = Normothermia, T = Torpor, A = Arousal).

		Per1	Bmal1	Rev-Erbα	Clock	Per2	MT1	MT2	GPR50	QR2
		Median ratio	Median ratio	Median ratio	Median ratio	Median ratio	Median ratio	Median ratio	Median ratio	Median ratio
Cerebral cortex	**T vs N**	1,54	0,81	0,81	0,75	1,24	1,23	0,83	2,09	1,04
	**A vs N**	2,22	0,78	0,91	0,94	2,88	1,16	1,56	3,04	0,80
	**A vs T**	1,44	0,96	1,11	1,25	2,31	0,95	1,87	1,45	0,76
Hypothalamus	**T vs N**	1,52	0,45	0,44	0,40	0,72	0,65	0,79	0,62	0,58
	**A vs N**	2,54	0,51	0,48	0,63	1,94	0,72	1,60	0,89	0,42
	**A vs T**	1,67	1,12	1,10	1,55	2,69	1,11	2,03	1,43	0,72
Pituitary gland	**T vs N**	3,48	1,22	1,38	0,86	2,42	0,68	0,91	0,61	0,86
	**A vs N**	2,80	3,16	0,83	0,98	3,16	0,74	1,42	1,09	0,76
	**A vs T**	0,80	2,59	0,60	1,14	1,31	1,08	1,56	1,79	0,88
Retina	**T vs N**	1,49	0,93	0,87	0,79	1,14	0,84	0,96	1,30	0,92
	**A vs N**	1,87	1,49	0,96	1,01	2,69	0,80	2,30	1,74	0,61
	**A vs T**	1,25	1,60	1,11	1,28	2,36	0,95	2,39	1,34	0,66
Liver	**T vs N**	2,74	1,51	1,02	0,92	3,13	0,50	0,28	0,46	1,04
	**A vs N**	2,25	1,14	0,58	1,01	3,81	0,55	0,37	0,53	0,71
	**A vs T**	0,82	0,75	0,57	1,10	1,22	1,10	1,31	1,13	0,68
BAT	**T vs N**	3,11	1,28	1,49	0,76	1,27	0,57	0,43	0,49	1,12
	**A vs N**	2,67	1,06	1,12	1,07	3,23	0,39	0,37	0,68	0,83
	**A vs T**	0,86	0,83	0,75	1,41	2,55	0,69	0,86	1,39	0,74
Heart	**T vs N**	3,72	1,08	1,08	0,88	2,04	1,25	0,63	1,05	1,19
	**A vs N**	3,69	1,14	0,59	1,13	7,59	1,00	0,67	0,95	1,07
	**A vs T**	0,99	1,06	0,54	1,29	3,72	0,79	1,06	0,90	0,90
Adrenal glands	**T vs N**	2,77	2,10	1,01	0,82	1,80	0,43	0,74	0,76	1,14
	**A vs N**	3,16	2,02	1,03	1,18	3,97	0,56	0,83	1,08	0,99
	**A vs T**	1,14	0,96	1,03	1,44	2,20	1,31	1,11	1,41	0,87
		**SIRT1**	**TXNIP**	**PPARα**	**PGC1α**	**FGF21**	**ObR**	**Dio2**	**Dio3**	**UCP1**
		**Median ratio**	**Median ratio**	**Median ratio**	**Median ratio**	**Median ratio**	**Median ratio**	**Median ratio**	**Median ratio**	**Median ratio**
Cerebral cortex	**T vs N**	0,83	1,21	0,61	0,50	0,95	1,11	0,67	0,96	
	**A vs N**	1,09	0,79	1,09	0,77	1,33	1,11	0,55	0,87	
	**A vs T**	1,30	0,65	1,79	1,55	1,40	1,00	0,82	0,90	
Hypothalamus	**T vs N**	0,49	0,68	0,27	0,29	0,64	1,04	0,50	0,89	
	**A vs N**	0,63	0,52	0,72	0,56	1,19	0,63	0,61	1,00	
	**A vs T**	1,30	0,76	2,73	1,91	1,84	0,61	1,23	1,13	
Pituitary gland	**T vs N**	1,05	1,46	0,93	1,02	2,18	0,70	0,40	0,70	
	**A vs N**	1,14	0,81	1,22	1,04	3,02	0,71	0,55	1,03	
	**A vs T**	1,09	0,56	1,32	1,02	1,39	1,01	1,39	1,47	
Retina	**T vs N**	0,84	1,42	0,37	0,62	2,18	0,73	0,53	0,67	
	**A vs N**	1,24	1,11	0,60	0,91	3,95	0,72	0,64	0,86	
	**A vs T**	1,47	0,78	1,62	1,46	1,81	0,99	1,21	1,29	
Liver	**T vs N**	1,51	1,68	0,88	1,77	0,19	2,71	0,37	0,90	
	**A vs N**	1,31	1,44	0,67	2,41	2,16	2,21	0,54	1,00	
	**A vs T**	0,87	0,86	0,76	1,36	11,35	0,82	1,47	1,11	
BAT	**T vs N**	0,87	0,93	0,71	0,81	0,60	0,93	0,30	0,82	1,10
	**A vs N**	1,46	0,87	0,48	1,50	0,78	0,60	0,23	2,34	0,80
	**A vs T**	1,69	0,93	0,68	1,85	1,29	0,65	0,76	2,84	0,72
Heart	**T vs N**	1,04	0,77	0,72	0,81	1,12	0,98	0,79	1,87	
	**A vs N**	1,17	0,40	0,80	0,73	0,90	0,69	0,37	2,11	
	**A vs T**	1,12	0,53	1,12	0,91	0,80	0,70	0,47	1,13	
Adrenal glands	**T vs N**	0,94	0,71	0,80	0,58	0,46	0,63	0,16	0,99	
	**A vs N**	1,33	0,98	1,22	1,08	2,21	0,51	0,16	1,98	
	**A vs T**	1,41	1,38	1,52	1,84	4,75	0,81	1,00	2,00	

### Organ-dependent gene profiling during hibernation cycles

**Figure 2:**
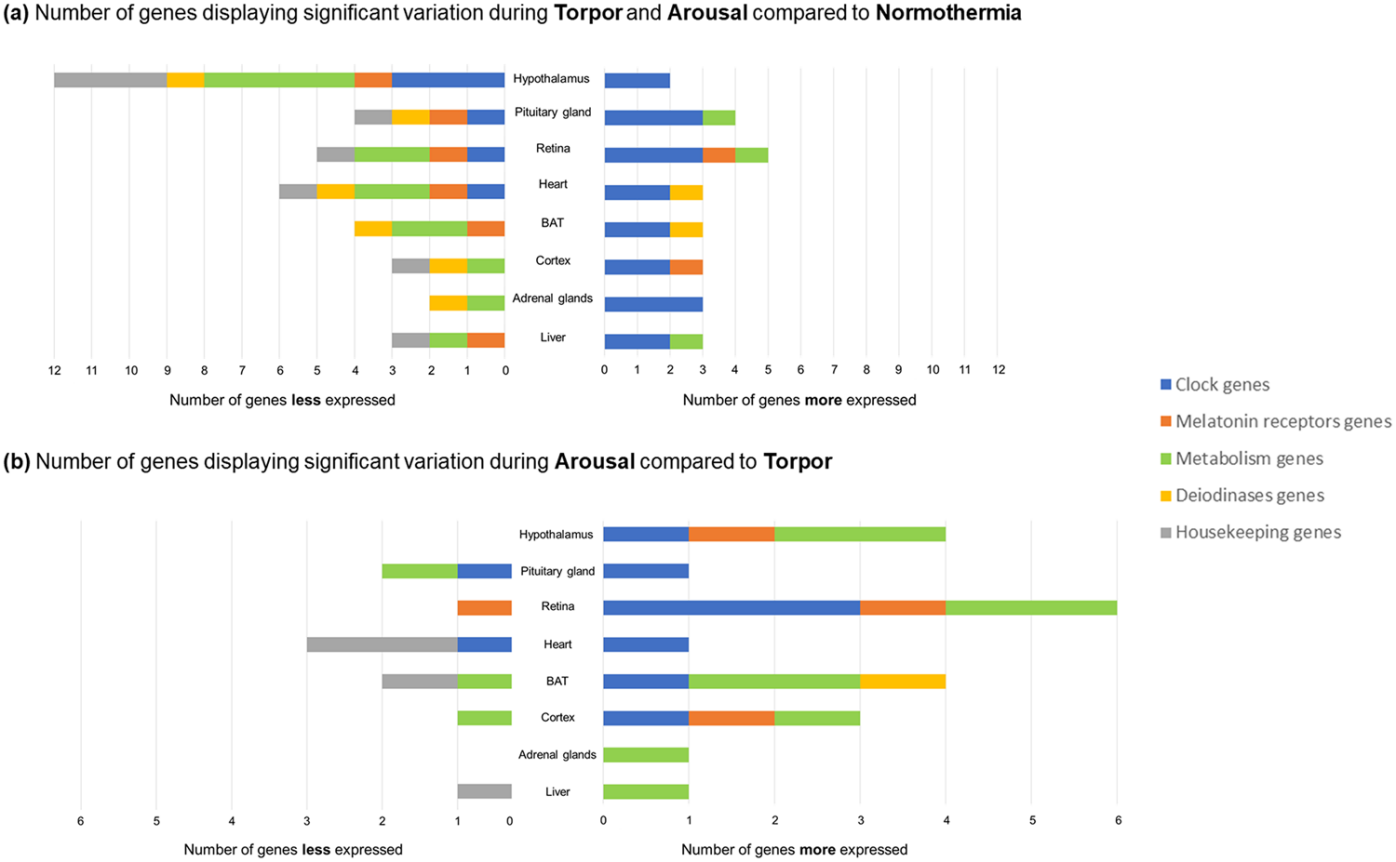
Changes in the expression of functional families of genes in various tissues of the European hamster according to hibernation stage. Data represent the number of genes per functional family that were down-regulated (left side) or up-regulated (right side) in eight central or peripheral tissues. (**a**) Gene expression during torpor and arousal compared to gene expression during normothermia. (**b**) Gene expression during arousal compared to gene expression during torpor.

In each central or peripheral organ, numerous genes were differentially expressed according to the hibernation phases (Fig. 2). Hypothalamus samples clearly contained the highest number of genes with significant differences in expression. Large numbers of differential genes were also found in other nervous/neuroendocrine organs, including the pituitary gland and the retina, but not the cortex. On the other hand, genes expressed in the liver and adrenal glands appeared to be the least affected.

Further examination of whether gene expression was increased or decreased in a given hibernation state revealed that more genes were down-regulated (39 genes) than up-regulated (26 genes) in torpor and arousal compared to in normothermia (Fig. 2a). Moreover, hypothalamus samples exhibited the highest number of down-regulated genes (12 genes) in torpor and arousal compared to normothermia, while only 2 genes were up-regulated. Most of the other organs showed similar numbers of up-regulated and down-regulated genes. A surprisingly low number of genes were differentially expressed when hamsters arose from torpor (Fig. 2b). Overall these results highlighted the strong alteration of hypothalamic genes during hibernation, with down-regulation of a strikingly large number of genes in torpor or arousal as compared to normothermia. During arousal, there is a moderate change in gene expression with the hypothalamus, the BAT and the retina showing the highest number of up-regulated genes.

### Profiles of functional groups of genes during hibernation cycles

To determine whether some functional groups of genes might be particularly relevant to the hibernation states, separate analyses were performed to establish the hibernation profiles of four categories of genes: clock system, melatonin receptors, thyroid hormone metabolism, and general metabolism.

#### Clock genes

**Figure 3:**
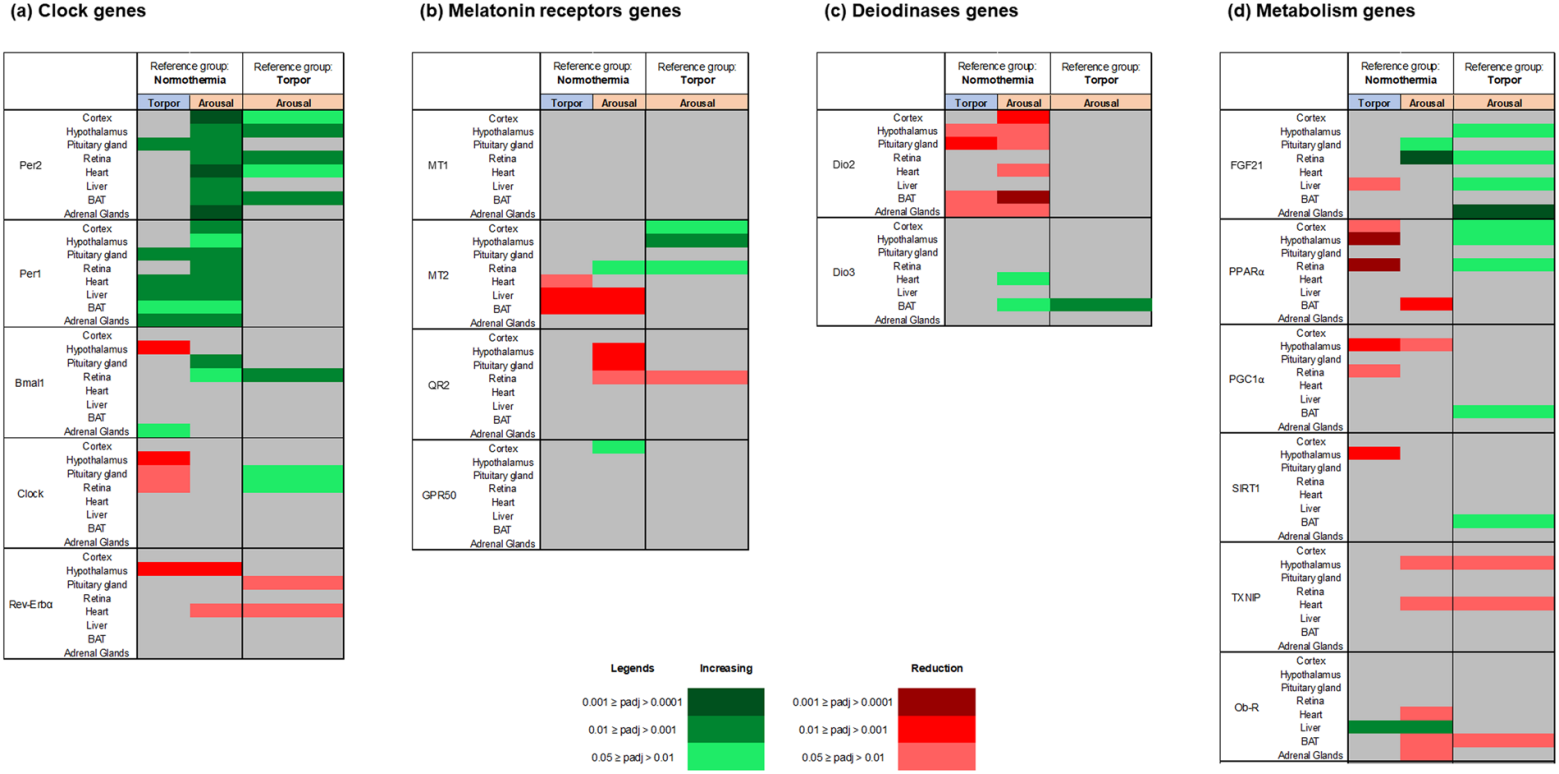
Changes in the expression of individual genes in various tissues of the European hamster according to hibernation stage. Genes are grouped according to their functional family: clock genes (**a**), melatonin receptor genes (**b**), deiodinase genes (**c**), and metabolism genes (**d**). In each table, the colour indicates significant up-regulation (green), down-regulation (red), or no change (grey) of the mRNA levels in the eight investigated tissues, after adjustment of their *P* values. Colour intensity is a function of the level of significance.

**Figure 4:**
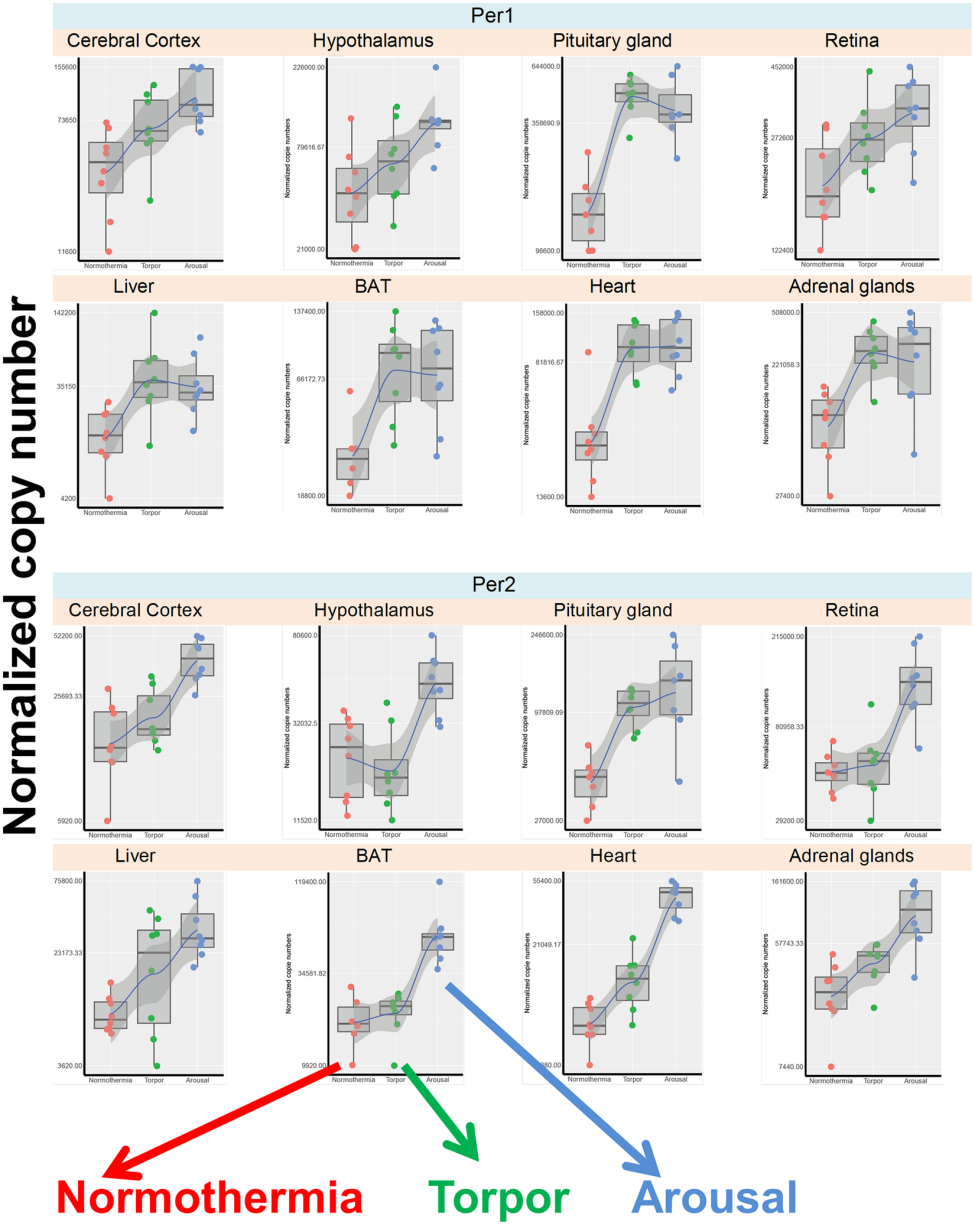
Boxplot presentation of mRNA values of the individual genes *Per1* and *Per2* across the three hibernation stages in all tissues. Individual samples values for each gene were represented, we also plotted a spline curve on those graphs (without a modulation purpose) to highlight the sense of gene variation across conditions. Normothermia in red, Torpor in green, Arousal in blue.

The Per1 and Per2 genes showed large differential expression among the hibernation phases in all organs (Fig. 3a) and these variations appeared not linked to sampling time for most organs (see material and method section). Per2 was strongly up-regulated during arousal compared to in the normothermia state in all investigated organs, and was up-regulated to a less extent during arousal compared to torpor in five organs, including the hypothalamus and BAT. Per1 expression was also markedly up-regulated during arousal in all analysed organs and, to a lesser extent, during torpor compared to during normothermia. In order to better visualize these variations in individual organs across the three hibernation stages, examples of boxplot are given in the Fig. 4. The other measured clock genes showed smaller variations. The Bmal1 mRNA level was higher in arousal compared to normothermia in the pituitary gland and retina, and was higher in arousal compared to torpor in the retina. Clock and Rev-Erbα were down-regulated during arousal and torpor compared to normothermia, particularly in nervous tissues for the Clock gene, and in the hypothalamus and heart for the Rev-Erbα gene. Notably, in the hypothalamus, all clock genes were either down-regulated or unchanged during torpor compared to normothermia, thus confirming the results of whole gene analysis (Fig. 2).

#### Melatonin receptors encoding genes

Genes encoding melatonin receptors displayed variations during the hibernation cycle that were strongly dependent on the receptor subtype (Fig. 3b). Notably, the MT_2_ receptor showed the largest variations, with decreased expression in torpor and arousal compared to in normothermia in peripheral organs (liver, heart, and BAT), but increased expression in arousal compared to torpor in central tissues. The gene encoding QR_2_ was down-regulated during arousal compared to normothermia in nervous tissue (hypothalamus, retina and pituitary). The orphan receptor *GPR50* showed altered expression during arousal compared to normothermia, only in the cortex. MT_1_ expression did not significantly vary during hibernation in any investigated organ.

#### Deiodinase genes

**Figure 5:**
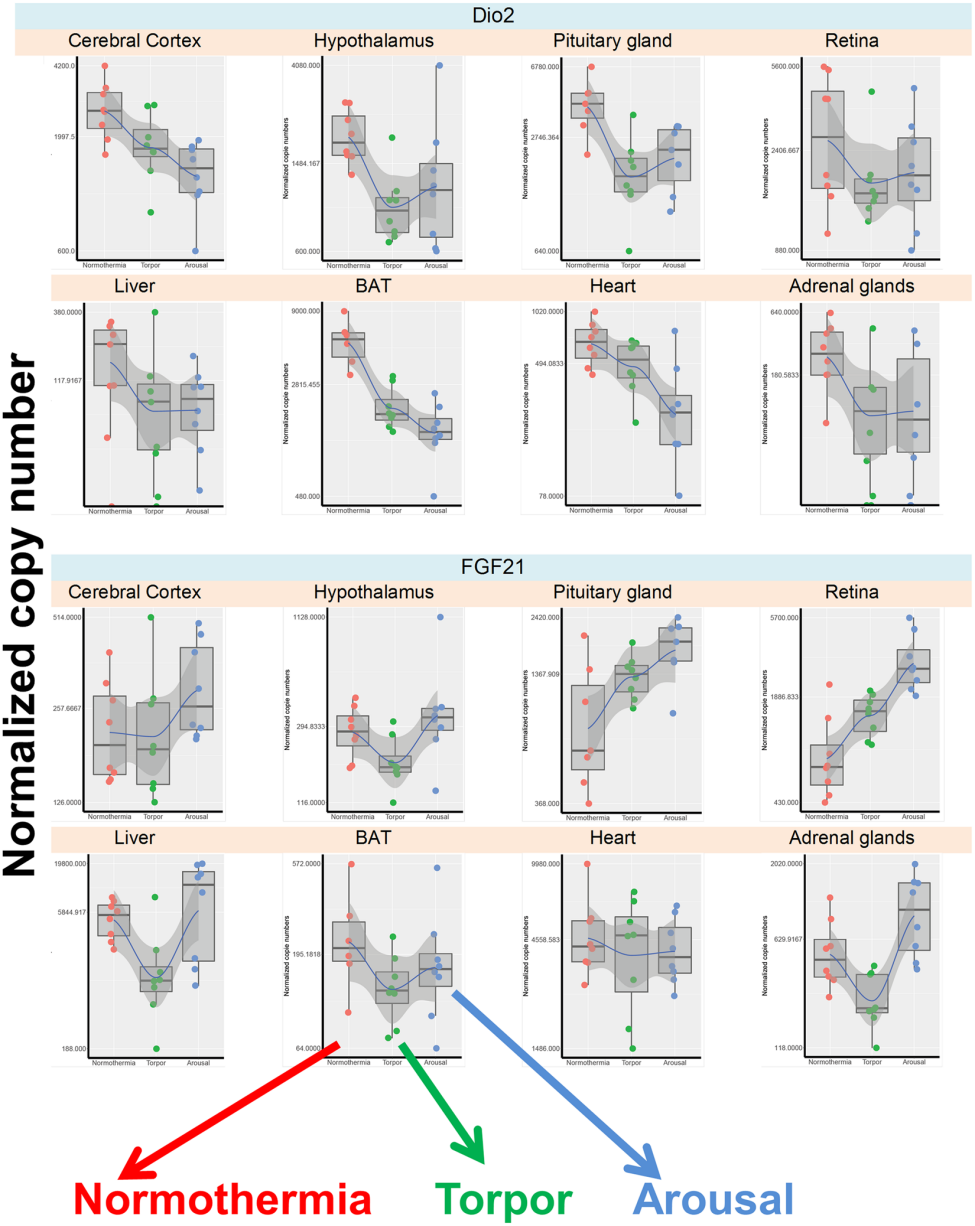
Boxplot presentation of mRNA values of the individual genes *Dio2* and *FGF21* across the three hibernation stages in all tissues. Individual samples values for each gene were represented, we also plotted a spline curve on those graphs (without a modulation purpose) to highlight the sense of gene variation across conditions. Normothermia in red, Torpor in green, Arousal in blue.

The genes encoding two enzymes involved in the thyroid hormone metabolism, Dio2 and Dio3, displayed different hibernation profiles (Fig. 3c). *Dio2* expression was markedly decreased in torpor and arousal compared to in normothermia, particularly in the hypothalamus, pituitary gland, BAT, and adrenal glands (Fig. 5). In contrast, *Dio3* expression was increased during arousal compared to during normothermia and torpor in the BAT, and in during arousal compared to normothermia in the heart. Notably in BAT during arousal, *Dio2* mRNA was down-regulated in association with up-regulation of *Dio3* mRNA, suggesting reduced thyroid metabolism.

#### Genes involved in general metabolism

The hibernation profiles of genes involved in general metabolism revealed large differences among genes and organs, making it difficult to illustrate a general scheme (Fig. 3d). A number of genes were down-regulated in torpor compared to normothermia, including *FGF21* in the liver, *PPARα* in central tissues, *PGC1α* in the hypothalamus and retina, and *SIRT1* in the hypothalamus. Overall in the hypothalamus *PPARα, PGC1α*, and *SIRT1* were down-regulated. In contrast, the peripheral organs unexpectedly showed no change in metabolic gene expression between torpor and normothermia, with the exception of *FGF21* down-regulation and *Ob-R* up-regulation in the liver. A number of genes were up-regulated in the arousal state compared to the torpor state, particularly *FGF21* in hypothalamus, retina, liver, and adrenal glands; *PPARα* in central tissues; and *PGC1α* and *SIRT1* in the BAT. Only *TXNIP* in the hypothalamus and heart, and *Ob-R* in the BAT were reduced in arousal compared to torpor. Unexpectedly, *UCP1* mRNA in the BAT did no vary according to the hibernation stage (not shown). In order to better visualize *FGF21* variations in individual organs across the three hibernation stages, examples of boxplot are given in the (Fig. 5).

#### Housekeeping genes

Although this is not required in the ddPCR approach, we examined classical housekeeping genes to determine whether they were generally altered due to the torpor conditions. Our data (not shown) indicated no organ-dependent or stage-dependant common variations among the three investigated genes: *tubulin*, *actin*, and *G6PD*. These observations indicated that there was no general alteration of gene expression according to hibernation stage in any investigated organ.

### Circulating hormones during the hibernation cycles

Some of the hormones related to the functional groups of genes—such as melatonin, T3, T4, glucose, insulin, and leptin—were assayed at the different hibernation states. Although the circulating levels of leptin tended to decrease during torpor, as reported for the Syrian hamster^50^, overall the circulating concentrations of these hormones did not significantly differ among the three hibernation states possibly due to high inter-individual variations (Supplementary Table S2). Thus, we were unable to examine correlations between changes in circulating hormone levels and changes in gene expression.

## Discussion

With the objective to better understand the mechanisms which may control hibernation, we examined the molecular signatures of a set of genes related to daily and seasonal timing, torpor regulation, and energy homeostasis, from eight different organs, in association with three hibernation phases during a winter-like heterotherm period in the well-established hibernating species, the European hamster.

Analysing the overall changes in functional families of genes revealed ubiquitous up-regulation of the core clock genes *Per1* and *Per2*, and to a lesser extent *BMAL1* and *Clock*, upon arousal from torpor. This up-regulation of clock genes is most likely associated with arousal from torpor rather than due to a shift in circadian fluctuations, since we previously determined that circadian clockwork stops oscillating in the European hamster SCN during torpor^30^ and in most genes we found no apparent, or weak, correlation between the sampling time and the levels of clock gene RNA (a strong correlation between time sampling and *Period* genes has been observed in the pituitary gland for the expression of *Per1* and in both retina and adrenal glands for *Per2* expression. In these organs, it is complicated to conclude about the effect of hibernation state on Period genes expression). For the other organs, the absence of correlation strongly suggests that the sampling time did not modify the expression of these genes within the time frame of our experimental setup. The *Per* gene up-regulation could be explained, among others, by the reported increase of glucocorticoids during arousal^51^, and correlation between *Per* gene expressions and glucocorticoid concentration should be measured to test this hypothesis. Our present results further showed an overall down-regulation of *Bmal1*, *Clock*, and *Rev-Erbα* in the hypothalamus of torpid hamsters, further indicating arrest of the circadian clock. The role of the master circadian clock during torpor remains a matter of discussion. Thirteen-lined ground squirrels exhibit markedly increased SCN activity during torpor and arousal, as indicated by c-fos expression^52,53^. Moreover, in Djungarian hamsters^54^ and ground squirrels^55^, SCN lesions do not prevent torpor states but only alter their timing. Altogether, these data indicate that the SCN clock is not essential for torpor occurrence but may play a crucial role in its temporal organization. Moreover, our findings indicate that *Per* genes may be essential for post-torpor re-entrainment of the clock.

Studies in various hibernating species report no night-time increase in melatonin during the torpor states, due to SCN clock arrest, with a subsequent rapid restoration of the nocturnal rhythm upon arousal^30,56–58^. Studies investigating changes in 2-^125^I-iodomelatonin binding on melatonin receptor density during hibernation in the brains of hedgehogs^59^ and ground squirrels^60^ have reported decreased numbers of binding sites in the *pars tuberalis* during torpor compared to normothermia. Our present data showed that the gene encoding MT_1_ was not differentially expressed according to hibernation phase in any of the investigated tissues. In contrast, MT_2_ gene expression increased during arousal in central organs (cortex, hypothalamus, and retina), and decreased during torpor and arousal in peripheral organs (heart, liver and BAT). A recent study investigated how the melatonin receptor antagonist luzindole may influence the brain of the hibernating ground squirrel, and demonstrated that melatonin receptor signalling promotes neuroprotection and optimizes mitochondrial function during arousal from torpor^61^. Therefore, the increased central MT_2_ expression observed upon arousal indicates that this receptor may be involved in the neuroprotective function of melatonin during hibernation. This function may be particularly relevant in the retina, which expresses high levels of MT_2_ and displays profound synaptic remodelling during hibernation^62–64^. Melatonin also reportedly activates arousal thermogenesis through peripheral actions^65–68^; however, our data did not reveal significant changes in MT_1_ expressions in any peripheral organs and a decrease of MT2 in the BAT. Mice bearing a mutation in the melatonin-related receptor GPR50 display enhanced propensity to fasting-induced torpor^17,69^, but we found that *GPR50* gene expression did not vary among the hibernation stages of the European hamster. Similarly, we found no variations in the expression of QR2 (the *MT*_*3*_ binding site^14^), suggesting either that the enzyme plays a minor role in this context, or that it is not regulated through variation of gene expression. Altogether, our data indicated that the gene encoding MT_2_ was most substantially altered according to the hibernation cycles, with opposite patterns in central and peripheral organs.

The adaptation to winter physiology, especially torpor, requires reduced availability of thyroid hormones, particularly T3^26,27,70,71^. We show here that the Dio2 enzyme (that converts T4 to T3) was decreased in central structures: hypothalamus and pituitary gland; and peripheral organs: BAT and adrenal glands, during torpor and arousal. The Dio3 enzyme (that degrades T3) was increased during torpor in the heart and BAT. These alterations lead to reduced T3 concentrations during torpor and arousal. Two recent studies in the Djungarian hamster demonstrated that increasing peripheral^72^ or intra-hypothalamic^27^ T3 resulted in reduction of torpor induction and torpor bout duration and depth, confirming the pivotal role of T3 in torpor regulation and strengthening our observation of reduced T3 metabolism during torpor.

Torpor induction is associated with a switch in energy utilization from carbohydrates to lipids^31^. In the present study, we followed the changes in expression of *FGF21* and its upstream regulator *PPARα*, since they reportedly play major roles in adaptive starvation responses and torpor promotion^38,39^. Indeed, during torpor, *FGF21* expression was decreased in the liver, and *PPARα* expression was decreased in the cortex, hypothalamus, and retina. Upon arousal, *FGF21* expression was increased in the hypothalamus, retina, liver, and adrenal glands; and *PPARα* expression was increased in the cortex, hypothalamus, and retina. *SIRT1* and *PGC1α* are involved in gluconeogenesis and thermogenesis^35,73^, and both were down-regulated in the hypothalamus during torpor, and up-regulated in the BAT during arousal, likely in response to increasing energy demand. TXNIP is involved in hypothalamic homeostasis and hepatic gluconeogenesis^40,41^, and is reportedly up-regulated in the hypothalamus during various types of torpor^42,43^. In our present analysis of the European hamster hypothalamus, TXNIP gene expression was not higher during torpor compared to normothermia, but was significantly reduced during arousal, possibly to adjust the central metabolic demand when the animals awoke from torpor. Altogether, our findings indicated that selected genes involved in metabolic pathways were generally down-regulated during torpor and up-regulated during arousal, particularly in the hypothalamus.

Comparison among the various investigated central and peripheral organs revealed that each organ displayed a specific hibernation gene profile, potentially indicating different functions of these organs during the hibernation cycle. In particular, the hypothalamus and BAT warrant closer analysis. These tissues are thought to play critical roles in hibernation, and previous studies reported thorough transcriptomic analysis at different hibernation stages.

Little is known about the genes responsible for the highly efficient fat burning activity that occurs during the hibernation arousal process. BAT generates heat, notably in newborns and hibernating mammals. BAT thermogenesis involves norepinephrine-dependent β-oxidation of free fatty acids under the control of the hypothalamus via the sympathetic nervous system. It appears that thyroid hormones are also involved in this process since T3 influences BAT thermogenesis either directly or indirectly through central sites. A recent analysis of BAT in the thirteen-lined ground squirrel demonstrates differential expression of 14% of the examined genes across four collection points throughout the year and hibernation stages^45^. Comparing the transcriptomes of torpid and aroused squirrels revealed that a few genes encoding transcription factors were significantly down-regulated during torpor, notably BHLHE40 which is involved in circadian rhythms^74^. Our analysis of European hamster BAT confirmed that *PGC1α* and *SIRT1* were up-regulated during arousal compared to torpor. We further observed that the *Dio2/Dio3* mRNA ratio decreased during arousal, indicating reduced local availability of T3 in BAT. In agreement with the increase in BHLHE40 in squirrel BAT, we observed higher expression of *Per* genes during arousal in European hamsters, supporting a putative role of BAT molecular clock machinery during arousal from torpor.

The hypothalamus coordinates a number of biological functions, including thermal and metabolic processes, circadian organization, sleep, reproduction, and the control of both hibernation and daily torpor^53,75^. During torpor, minimal functional brain activity persists to prevent nervous tissue damage and to allow the animals to rapidly and completely recover from hypothermia. Recent studies involving RNASeq analyses of the hypothalamus and cerebral cortex from thirteen-lined ground squirrels (true hibernator)^43^, and of the hypothalamus from Djungarian hamsters (daily torpidators)^48^, at various stages of torpor or interbouts have reported different strategies implemented in the hypothalamus and cerebral cortex during hibernation. In the hypothalamus, the differentially expressed genes are involved in protection against DNA damage, protein turnover through ubiquitination, feeding and satiety signalling, seasonal timing mechanisms, and fuel utilization. In the cerebral cortex, the candidate genes are involved in synapse remodelling and plasticity. Among Djungarian hamsters entering torpor, about 1% of the 27,830 identified genes in the hypothalamus are differentially expressed, most of which are involved in metabolic and cellular functions. The majority of the top 20 most down-regulated genes encoded transcription factors, which may be responsible for the general suppression of protein synthesis during torpor^48^. In our study, the European hamster’s hypothalamus showed the greatest number of candidate genes that were differentially expressed during the hibernation stages. Most were down-regulated during torpor/arousal compared to normothermia, particularly genes involved in metabolic processes (*PPARα*, *PGC1*, *SIRT*, *TXNIP*, and *Dio2*) and genes involved in the core circadian clock (*Bmal1*, *Clock*, and *Rev-erbα*). During arousal from torpor, we observed up-regulation of some metabolic (*PPARα* and *FGF21*) and circadian (*Per2*) genes, as well as *MT*_*2*_. Altogether, our data support the metabolic silencing of the hypothalamus during torpor, and suggest the involvement of new timing components in the hibernation processes.

Studies using various transcriptomic approaches have indicated that a rather limited number of genes are differentially expressed during hibernation, depending on species, organs, and hibernation stages. Thus, it appears likely that posttranscriptional and posttranslational mechanisms are also involved in the dramatic changes in body temperature and in other physiological variables that accompany hibernation. A recent large-scale proteomic analysis also revealed a relative stability of the proteome throughout the extreme physiological changes of hibernation^3^. Most of the observed protein changes were linked to seasonal changes, i.e. between the homeothermic and heterothermic periods. If even some of the limited differences in protein abundance according to torpor/arousal cycles are attributed to protein modifications, this indicates that the total protein pool is extremely stable across the hibernation season, despite prolonged fasting and wide variations in metabolic levels. This stability is not completely unexpected, since the profound temperature-mediated suppression of both transcription and translation machinery during torpor drastically limits the ability to synthesize new gene products. Across the available proteomic screens of hibernator tissues, cytoskeleton regulation was the most consistent signal, possibly associated with the temperature-induced neural retraction and cytoskeletal depolymerisation. Altogether these results suggest that the most dramatic protein changes associated with the torpor/arousal cycle may be due to post-translational modifications. Notably, protein acetylation appears to be a key component of the torpor/arousal switch^3,76^.

Hibernation is a key process enabling animals to survive harsh natural conditions. Identifying the complex molecular mechanisms orchestrating the extreme changes in hibernation could provide important contributions to the development of therapeutic strategies to improve medical outcomes in a number of conditions, including hypothermic injury, organ transplantation, stroke recovery, cardiac arrest, muscle disease, and other ischemia/reperfusion insults. Numerous studies have investigated how torpid animals tolerate cerebral ischemia following dramatic reductions of blood flow and oxygen concentration, display reversible rapid and pronounced synaptic flexibility in which synapses retract during torpor and rapidly re-emerge upon arousal, and retain skeletal muscle mass despite prolonged immobilization and lack of nutrition^77–79^. There remains much to be learned about the cellular, molecular, and systems-wide mechanisms that protect heterothermic mammals during torpor/arousal cycles, which may guide discovery of new therapeutics.

Strikingly, the previously performed transcriptomic, proteomic, and metabolic studies indicate large inter-species and inter-tissue differences in the involved genes and proteins, highlighting the importance of analysing different hibernating species. Our present study is the first to report gene profiling during torpor/arousal cycles in several organs of the European hamster. We report a broad reduction in our candidate gene expression, especially those involved in metabolic activity, within the hypothalamus of torpid hamsters, and a decrease in the genes of thyroid hormone synthesis, notably in the BAT, during the torpor/arousal stages. Furthermore, we observe a general up-regulation of the core circadian clock genes *Per1* and *Per2* upon arousal, suggesting a possible resetting of body clocks at the start of the active period.

## Methods

Animal experimentation was conducted in accordance with the French National Law implementing the European Communities Council Directive 2010/63/EU and the French Directive 2013-118. Animal procedures were reviewed by the local ethical committee (Comité Régional d’Ethique en Matière d’Expérimentation Animale de Strasbourg, CEEA 35) and the official authorization was given on July 2015 by la direction générale de la recherche et de l’innovation under the number #01546.02.

### Animals

European hamsters (*Cricetus cricetus)* are a well-established hibernating species^80^. The adult male hamsters used in this study were bred in-house (Chronobiotron, UMS 3415). Animals were individually maintained in cages, and food and water were provided *ad libitum* throughout the study. At the start of the experiment, each hamster was administered isoflurane anaesthesia, and intraperitoneally implanted with a Thermochron iButton (DS1922L; Maxim, Dallas, TX) to monitor its body temperature.

**Figure 6:**
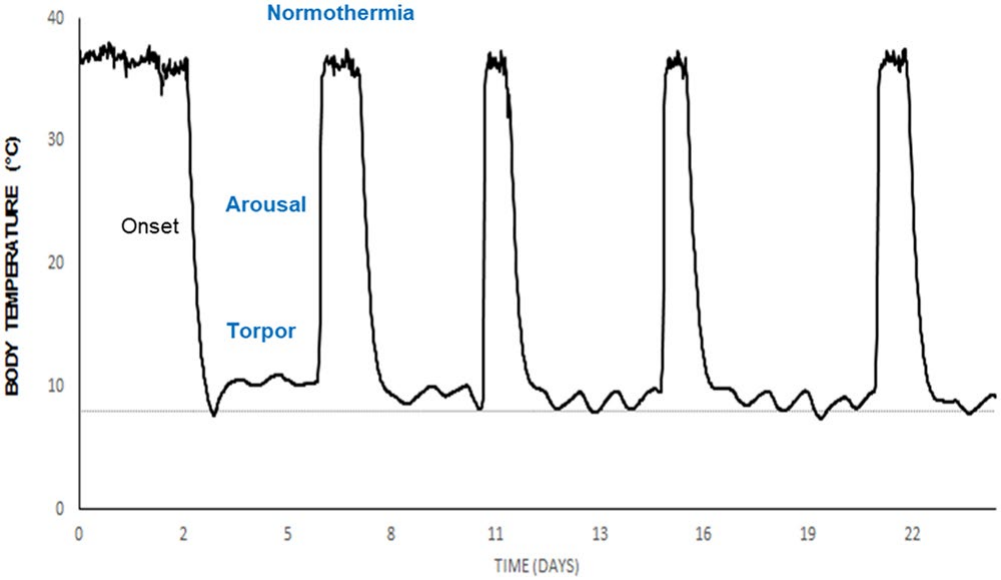
Typical hibernation pattern of a male European hamster. The graph shows body temperature variations, measured by intra-peritoneal iButton, over 25 consecutive days in a hamster maintained with a short photoperiod (10 hours of light and 14 hours of dark) at an ambient temperature of 8 °C (dashed line). Tissues were sampled at three different phases of a characteristic hibernation cycle, as determined by body temperature: torpor (8–10 °C), arousal (21–31 °C), and normothermia (33–36 °C).

From July 6^th^ to September 14^th^ 2015, 24 hamsters were subjected to a short photoperiod (SP) comprising 10 hours of light and 14 hours of dark, with the lights on at 8:00 (Zeitgeber ZT0). This induced a winter phenotype characterized by low body weight (mean, 375.6 ± 12 g) and complete gonadal regression confirmed by scrotal palpation. Starting on September 15^th^, the animals were transferred to a climatic room maintained at 8 °C to induce deep torpor bouts characteristic of European hamster hibernation (Fig. 6). Individual hibernating behavior was followed daily for 3 weeks, after which the animals were euthanized by CO_2_ inhalation. The animals were euthanized during three different phases of hibernation bouts (n = 8/group): normothermia (stable high Tb of ~36 °C for at least 3 days), torpor (stable low Tb of ~9 °C for at least 3 days), or arousal (increasing Tb between 21–36 °C, 2 hours after the beginning of arousal from torpor). The experiment ended on October 28^th^. In order to reduce bias related sampling times, we have limited as much as possible the time-window of euthanasia. Hamsters from the normothermia and torpor groups were all sampled between ZT2 and ZT6, but sampling of aroused hamsters was more delicate to control as the objective was to euthanize hamsters exactly 2 hours after the starting of the arousal. We tried to overcome this issue by promoting a gentle hand-warming arousal of the animals at ZT0/ZT1 and looking at their awakening behaviors, however animals started their arousal with different delays and therefore the sampling of this group occurred between ZT4 and ZT9.5. Thus, in order to investigate the potential effect of sampling times on genes most susceptible to variations (*Per1* and *Per2*), we calculated the non-parametric Spearman correlation coefficient for the arousal phase (a non-parametrical method was chosen to be in accordance with the non-normal distribution of ddPCR data.) between time of sampling *versus Per1* and time sampling *versus Per2* mRNA quantity for each organ. We observed that in most organs *Per1* and *Per2* expression is not, or weakly correlated, to the time sampling (Spearman correlation coefficient for *Per1*: cerebral cortex = −0.24; hypothalamus = −0.02; Pituitary gland = −0.91; retina = −0.17; liver = 0; BAT = −0.48; heart = −0.69; adrenal glands = −0.43. *Per2*: cerebral cortex = 0.38; hypothalamus = 0.29; pituitary gland = 0.07; retina = 0.81; liver = −0.21; BAT = −0.6; heart = −0.62; adrenal glands = −0.76). We observed strong correlation between time sampling and *Per1* expression in the pituitary gland and between time sampling and *Per2* expression in both retina and adrenal glands. Due to these correlations, it is difficult to conclude on the effect of hibernation state on *Period* genes expression for these organs. For the other organs, the absence of correlation strongly suggests that the sampling time did not modify the expression of these genes within the time frame of our experimental setup.

Blood was collected and centrifuged at 1,500 *g* for 15 min, and then the plasma was stored at −20 °C until hormonal assay. Eight central and peripheral organs—namely, the cerebral cortex, hypothalamus, pituitary gland, retina, liver, heart, brown adipose tissue, and adrenal glands—were rapidly removed, rinsed in cold Ringer’s solution, frozen in liquid nitrogen, and then stored at −80 °C until gene analysis.

Prior to this study, a preliminary experiment was performed with fewer animals per group, allowing adjustment of the procedure and selection of tissues and genes of interest. Although the results were generally similar, only the data from the second (2015) experiment are presented in this manuscript, due to their greater statistical strength.

### RNA extraction

Total RNA was extracted using the RNeasy® Lipid Tissue Mini Kit (Qiagen, Valencia, CA, USA) following the manufacturer’s protocol. Briefly, a sample of each organ (100 mg when possible) was added to 1 mL QIAzol Lysis Reagent, along with one 5-mm stainless steel bead (Qiagen, Valencia, CA, USA). The tissue samples were disrupted, and homogenized for 2 min at 20 Hz using the tissue lyser. Then the homogenates were incubated at room temperature (RT) for 5 min, followed by addition of 200 µL chloroform (Sigma, St. Louis, USA). Next, the homogenates were centrifuged at 12,000 *g* for 15 min at 4 °C. The upper aqueous phase containing RNA was transferred to a new tube and the final step was performed using the QIAcube protocol: RNeasy lipid Animal tissue (Qiagen, Valencia, CA, USA). RNA quality was measured using Agilent’s 2100 Bioanalyzer system according to manufacturer’s protocol (Agilent, Santa Clara, USA). RIN values are all presented in the Table 3. Total RNA was stored at −80 °C until use.

**Table 3:** The RIN of each sample was determined using Agilent’s 2100 Bioanalyzer system.

Hibernation phases	Animals	Cerebral cortex	Hypothalamus	Pituitary gland	Retina	Liver	BAT	Heart	Adrenal glands	Mean	SD
Torpor	HE15	6,9	7,8	9,1	9,7	8,7	8,3	8,7	9,8	8,8	0,8
	HE16	8,5	8,7	9,3	9,3	8,9	8,5	8,6	9,8		
	HE17	7,5	8,8	9,4	9,8	8,8	9	8,6	9,8		
	HE24	9,2	8,6	9,7	9,6	8,7	8,8	9,1	10		
	HE26	8,7	9,3	9,5	10	6,7	8,5	8,2	10		
	HE10	7	8,2	9,2	9,4	8,2	8,4	8,6	8,7		
	HE31	9,1	8,5	9,8	9,7	7,8	8,8	9,1	9,9		
	HE34	8,9	7,8	8,5	9,4	8	7,6	8	9,7		
Normothermia	HE9	8,4	8,7	9,6	9,8	8,3		8,8	9,8	8,7	0,7
	HE11	8,3	7,9	8,1	9,9	8,2	8,7	8,3	9,4		
	HE12	7,9	7,7	9	9,7	7,4	7,8	7,9	9,7		
	HE19	8	7,9	8,9	9,3	7,4	8,2	8	9,6		
	HE20	8,4	9	9,2	10	7,4	8,5	8,6	9,7		
	HE23	8,4	8,6	9,4	9,5	8,3	8,8	8,3	9,9		
	HE30	8,4	8,5		9,3	8,6	8,5	8,7	8		
	HE33	8,5	8,9	9,1	9,8	8,2		8,7	10		
Arousal	HE8	8,9	7		9,6	9	8,6	8,7	9,6	8,8	0,8
	HE14	8,6	8,3	9,5	9,8	6,9	8,1	8,4	9,7		
	HE18	8,7	7,8	7,7	8,6	8,6	8,6	8,9	9,8		
	HE21	8,4	8,9	8,9	9,9	8,3	7,9	8,1	8,9		
	HE25	8,1	8,2	8,8	9,9	7,6	8,4	8,1	9,7		
	HE27	9,2	8,9	9,6	10	7,8	8,3	8,3	10		
	HE28	9	9,2	9,5	9,9	8,6	8,3	9,1	9,8		
	HE29	8,4	9	8,3	9,6	7,5	8	8,4	9,9		
	**Mean**	8,39	8,43	9,10	9,65	8,08	8,39	8,51	9,63		
	**SD**	0,60	0,57	0,54	0,32	0,63	0,35	0,36	0,47		

### Cloning genes of interest

Since few *Cricetus cricetus* gene sequences are published *(Database Resources of the National Center for Biotechnology Information)*, the genes of interest were partially sequenced (about 500 bp) to enable the design of specific ddPCR assays.

European hamster hypothalamus total RNA (5 µg) was converted into cDNA using oligo dT primers and the PrimeScript™ High Fidelity RT-PCR Kit (Takara Bio USA, Mountain View, USA) following the manufacturer’s instructions. The polymerase chain reaction (PCR) was performed using Q5 High-Fidelity DNA Polymerase (NEB, Ipswich, MA, USA) following the manufacturer’s protocol. The 50-µL reaction mix comprised 1 µL template cDNA, 5 × Q5 reaction buffer, 10 mM dNTPs, 10 µM forward primer, 10 µM reverse primer, Q5 high-fidelity DNA polymerase 0.02 U/µL, and 5 × Q5 high GC enhancer. Sequences extracted from the National Center for Biotechnology Information (NCBI) were used to design 21 gene-specific primer pairs (Table 4). Amplicons were separated in 1% agarose gels stained with ethidium bromide, and the gel bands were visualized using U Genius (Syngene, Frederick, USA). If multiple bands were observed, the PCR products were purified using the high pure purification kit (Roche Mannheim, Germany). The eluted DNA was inserted into a blunt pJET vector using the CloneJET PCR cloning kit (Thermo Fisher Scientific, Waltham, USA), and transformed into DH10β chemically competent *Escherichia Coli* cells (NEB, Ipswich, MA, USA).

**Table 4:** Sequences of the forward and reverse primers used to clone European hamster genes of interest.

Genes	Reference sequences for primers design^a^	Forward Primer Sequences^b^	Reverse Primer Sequences^c^	*Cricetus cricetus* GenBank Accession Numbers^d^
*Per1*	NM_001034125.1: *Rattus norvegicus* period circadian clock 1 (Per1)	TGTGCACCCCTGGAGCCGCAAGG	TTTCTTGGCCCCCACAGGAACTG	MG598318: [*Cricetus cricetus*] period circadian clock 1 (Per1) mRNA, partial CDS
*Bmal1*	AB012600: *Rattus norvegicus* mRNA for BMAL1b	TAAAACGGATATAACCCCTGGGC CTGCCCTCTGGAGAAGGTGGCCC	TCTGGTTCCCCCTGGAATGCCTG ACCCAGCCCCGCATCTGCTTCCA	MG598320: [*Cricetus cricetus*] BmalI aryl hydrocarbon receptor nuclear translocator like (Arntl) mRNA, partial CDS
*Rev-Erbα*	XM_003498212.2: *Cricetulus griseus* nuclear receptor subfamily 1, group D, member 2 (Nr1d2)	GCTCTAACTCTGATGCCAACGG	GCTTTTGAGGTTTTCTTGCTCCAG	MG598307: [*Cricetus cricetus*] Nuclear receptor subfamily 1 group D member 2 (Nr1d2) mRNA, partial CDS
*Clock*	XM_016980269: *Cricetulus griseus* clock circadian regulator (Clock)	TCAATTGTTGACAGAGATGACAGTAG	TCTATTGTTCCTCGAAGCATGTGAC	MG598315: [*Cricetus cricetus*] circadian locomotor output cycles protein kaput (Clock) mRNA, partial CDS
*Per2*	XM_007622995: *Cricetulus griseus* period circadian clock 2 (Per2)	ACTGTGATGACAATGGGAAGGAGCT	ATGGAGGCAACTTGGTTAGAGATGT	MG598316: [*Cricetus cricetus*] Period circadian protein 2 (Per2) mRNA, partial CDS
*MT*_*1*_	U14110.1: *Phodopus sungorus* melatonin receptor (Mel-1a)	ATGAAGGGCAATGGTAGCACTCTGCTCAATGCC	CCGTATATAATTGCATTGAGGCAGCTG	MG598322: [*Cricetus cricetus*] Melatonin receptor 1 A (Mtnr1a) mRNA, complete CDS
*MT*_*2*_	NM_145712.2: *Mus musculus* melatonin receptor 1B (Mtnr1b)	TTGTTTGTGGTGAGTCTGGTCTTGG	GCCCATAGACAATGACGTTAAGGCAG	MG598323: [*Cricetus cricetus*] Melatonin receptor 1B (Mtnr1b) mRNA, partial CDS
*GPR50*	XM_007631612: *Cricetulus griseus* G protein-coupled receptor 50 (Gpr50)	CCGAACTGGCTGTATCTTGCAG	TCATACAGCCATCTCATCAGAA	MG598317: [*Cricetus cricetus*] G protein-coupled receptor 50 (Gpr50) mRNA, partial CDS
*QR2*	XM_007638944.1: *Cricetulus griseus* NAD(P)H dehydrogenase, quinone 2 (Nqo2)	TGGCAGGTAAGAAAGTGCTCATC/TCACTGGTTCCCTCTCTAATCCTG	TCTTCAGCCGCTTCGCCCATGATGC/TCTTCAGCCGCTTCGCCCATGATGC	KT992792: [*Cricetus cricetus*] NAD(P)H dehydrogenase quinone 2 mRNA, partial CDS
*Tubulin*	NM_001243978: *Cricetulus griseus* tubulin, alpha 1 A (Tuba1a)	ACACCTTCTTCAGTGAGACAGGCG	CCCAAAGATGTCAATGCTGCC	MG598321: [*Cricetus cricetus*] tubulin alpha 1B (Tuba1b) mRNA, partial CDS
*Actin*	NM_001244575: *Cricetulus griseus* actin beta (Actb)	CCCATTGAACACGGCATTGTC	CGACATCCGCAAAGACCTCTATG	MG598319: [*Cricetus cricetus*] actin beta (Actb) mRNA, partial CDS
*SIRT1*	XM_005070811.1: *Mesocricetus auratus* sirtuin 1 (Sirt1)	GTCATAGGTTAGGTGGTGAATATGCC	CACAGGAACTAGAGGATAAGATGTCGTC	MG598314: [*Cricetus cricetus*] sirtuin 1 (Sirt1) mRNA, partial CDS
*TXNIP*	XM_003498621.2: *Cricetulus griseus* thioredoxin interacting protein (Txnip)	CGACTCAGGAGGCAAAGAAAAAC	CAATCACCAGGGGAAGGTCAAG	MG598310: [*Cricetus cricetus*] Thioredoxin interacting protein (Txnip) mRNA, partial CDS.
*PPARα*	XM_007621010.1: *Cricetulus griseus* peroxisome proliferator-activated receptor alpha (Ppara)	GAATAAGTGCCAATACTGCCGC	CATACGCTATCAGCATCCCGTC	MG598312: [*Cricetus cricetus*] Peroxisome proliferator-activated receptor alpha (Ppara) mRNA, partial CDS
*PGC1α*	XM_007620649.1: *Cricetulus griseus* peroxisome proliferator-activated receptor gamma, coactivator 1 alpha (Ppargc1a)	TTTGATGTGTCGCCTTCTTGC	GGTGTAACGGTAGGTGATGAAACC	MG598311: [*Cricetus cricetus*] Peroxisome proliferator-activated receptor gamma, coactivator 1 alpha (Ppargc1a) mRNA, partial CDS.
*FGF21*	XM_007638697.1: *Cricetulus griseus* fibroblast growth factor 21 (Fgf21)	TGGACTGGATGAAATCTGGAGTTG	AAGGTCCCACCATGCTCAGTGG	MG598309: [*Cricetus cricetus*] Fibroblast growth factor 21 (Fgf21) mRNA, partial CDS
*Ob-R*	XM_007632623.1: *Cricetulus griseus* leptin receptor (Lepr)	GCCTGTCTTTCCAGAGAATAACCTTC	CGGCACTCACTTTACTCATTGGC	MG598308: [*Cricetus cricetus*] Leptin receptor (Lepr) mRNA, partial CDS
*UCP1*	NM_001281332.1: *Mesocricetus auratus* uncoupling protein 1 (Ucp1), mRNA	TCTACGATACTGTCCAGGAGTACTTC	CAGTCCACCGTCTGCCTCGACT	MG598313: [*Cricetus cricetus*] Uncoupling protein 1 (UCP1) mRNA, partial CDS

To obtain the sequences of the unpublished genes of interest, forward^b^ and reverse^c^ primers were designed using published sequences^a^. These sequences have been submitted to GenBank^d^.

Forward and reverse sequencing reactions were performed using the BigDye Terminator Cycle Sequencing Ready Reaction Kit (Applied Biosystems, Life Technologies Corporation, Carlsbad, CA, USA) using vector primers for amplification. Sequencing products were purified using the BigDye XTerminator® Purification Kit (Thermo Fisher Scientific, Waltham, USA), and analysed using an ABI 3730 XL Automated Sequencer (Applied Biosystems). Data analysis was performed using Sequencher® version 5.4.6 DNA sequence analysis software (Gene Codes Corporation, Ann Arbor, MI, USA). The *Cricetus cricetus* sequences of the nineteen non-published genes of interest were partially cloned and sequenced, and the results have been submitted to GenBank (Table 4).

### Digital droplet PCR

Primers and probes for the digital droplet PCR assay were designed using the Universal Probe Library (UPL) assay design centre: https://lifescience.roche.com/en_fr/brands/universalprobe-library.html (Roche Mannheim, Germany). Previously cloned *Cricetus cricetus* sequences were used as references. For technical reasons, custom assays from Biorad (Hercules, CA, USA) were used for the *clock* and *fgf21* genes.

**Figure 7:**
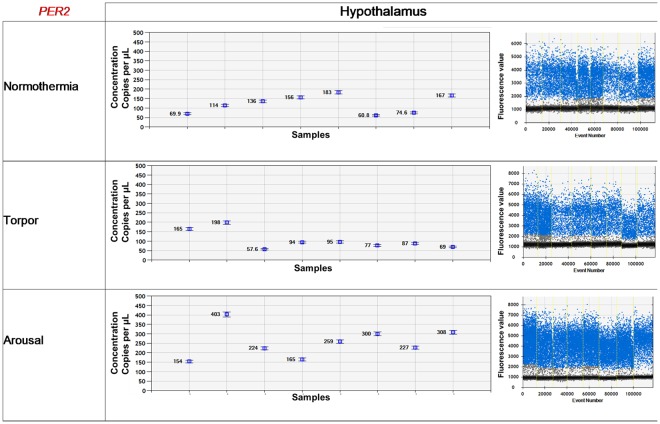
Typical example of digital droplet PCR (ddPCR) mRNA quantification. Using ddPCR, the exact number of *Per2* mRNA copies was quantified in hypothalamus tissue samples obtained from European hamsters at three different stages of the hibernation cycle (n = 8 from each stage): normothermia, torpor, and arousal. The graph on the left side depicts the calculated concentration of *Per2* mRNA copies/µL for each animal in each hibernation stage. The graph on the right side shows the fluorescence intensity of droplets in terms of the droplet number for each sample. QuantaSoft software was used to calculate the numbers of droplets containing *Per2* mRNA (blue) or not containing *Per2* mRNA (grey) in each sample.

RNA samples were directly partitioned using the One-Step RT-ddPCR Advanced Kit for Probes (Biorad, Hercules, USA). One-step RT-PCR reactions were carried out in a total volume of 22 µL, including 2–3 µL RNA (based on the RNA concentration), 5.5 µL super mix, 2.2 µL reverse transcriptase (20 U/µL), 1.1 µL DTT (300 mM), 0.6 µL forward and reverse primers, 0.55 µL probes, and molecular grade RNAse-free water (Table 5). Droplet generation was performed using an automated droplet generator (Biorad, Hercules, CA, USA) following the manufacturer’s recommendations. The thermal cycling conditions comprised 60 min reverse transcription at 50 °C, and 10 min enzyme activation at 95 °C; followed by 40 cycles of denaturation at 95 °C for 30 sec, and extension at the specific gene annealing temperature for 70 sec (Table 5); and finally 10 min enzyme deactivation at 98 °C using the CFX96 touch real-time PCR detection system (Biorad, Hercules, CA, USA). All steps used a ramp rate of 2 °C/sec. Subsequently, the droplets were analysed using the QX200 droplet reader (Biorad, Hercules, CA, USA). Figure 7 shows an example of ddPCR results for Per2 mRNA data measured in the hypothalamus. Each droplet from the sample is plotted on a 1-D graph of fluorescence intensity versus droplet number.

**Table 5:** Primer/probe sequences, RNA quantity, and annealing temperatures for digital droplet PCR (ddPCR).

Genes	Probes^a^	Probe References^b^	Forward Primer Sequences^c^	Primer Reverse Sequences^d^	RNA Quantity (ng)^e^	Annealing temperature (°C)^f^
*Per1*	UPL 63	4688627001	CCAGCACAACAAAGCGTAAA	TCAGAGGCTGAGGAAGCAGT	1	57
*Bmal1*	UPL 56	4688538001	CCAACCTTCCCACAGCTTAC	CCTGGAATGCCTGGAACA	5	57
*Rev-Erbα*	UPL 150	4694368001	TGTCTGTCAGTGGGAATGTCA	CTCTGTTTCTCACGCTTAGGAAT	1	57
*Clock*	AATGAAGTTACACTCTCAGATACAT	NA	CCACAAGATCAGATGGTA	TAGCGATCATGACAGATG	50	51
*Per2*	UPL 161	4694481001	CTTCTTGTCTGCAGGGAGGT	TGTCCTTATCAGTTCTTTGTGTGC	10	55
*MT*_*1*_	UPL 145	4694317001	CCCTCTGCTACGTGTTCCTG	GAGTTCCGGTTTGCAGATTG	150	59
*MT*_*2*_	UPL 131	4694155001	TGTGGTGAGTCTGGTCTTGG	AGGATCAGTGGGTAAGGGTACA	100	57
*GPR50*	UPL 86	4689119001	GCTGGCTCTTCCTCTAAGCA	GGCTGGTAGCAGGCTTAATG	150	55
*QR2*	UPL 68	4688678001	AAGACAGCTCTGACCAGTGACA	CTAGATCAGCTTCTTGCACCTTC	5	59
*Tubulin*	UPL 78	4689011001	GAGCGGCTCTCTGTCGATTA	GGGGCTGGGTAGATGGAG	0.01	59
*G6PD*	UPL 30	4687639001	TGTGGCAAAGCCCTGAAT	TGCCACATCTCGGAACTGTA	5	55
*Actin*	UPL 9	4685075001	GCTATGAGCTGCCTGATGG	GGCTGGAAAAGAGCCTCA	0.1	57
*SIRT1*	UPL 68	4688678001	GAAAGTGCTGGCCCAATAGA	GATTACCATCAAGCCGCTTACTA	5	57
*TXNIP*	UPL 125	4693604001	CCTTGCTGATCTATGTTAGTGTCC	TCACCAGGGGAAGGTCAA	1	57
*PPARα*	UPL 56	4688538001	CGGTGTGTATGAAGCCATATTC	ATCAGCATCCCGTCTTTGTT	1	57
*PGC1α*	UPL 41	4688007001	GTAGGCCCAGGTATGACAGC	CCTTTCAGATTCCCGTTTCTC	1	57
*FGF21*	ACACTGAAGTCCACCTGG	NA	ACCTCTACACAGATGACA	GGTTGTTGGCAAAGAAC	100	58
*Ob-R*	UPL 113	4693477001	CGCAGGAGATCAGACCAATC	ATTGATGGCCAGAACCGTAA	100	57
*Dio2*	UPL 22	4686969001	CCACCTTTCACTAGGCAACTG	AGTCGGCCACTGATGAGAAC	10	57
*Dio3*	UPL 135	4694198001	GCACCTAACTCGGAGGTCAT	ATAGTCGAGGATGCGCTGTC	100	57
*RBM3*	UPL 65	4688643001	TGGAAGCGGAAGATATGACA	TCTCTGGACCGCCCATATC	1	57
*UCP1*	UPL 21	4686942001	GGCAACCTACTGAGGTCGTG	ATCGGGGTTTGATCCCATA	0.1	57

Each ddPCR reaction requires probes^a,b^, forward^c^ and reverse^d^ primers, and determination of the optimal RNA quantity^e^ and annealing temperature^f^ were determined.

### Hormone analysis

Circulating hormone concentrations were determined by radioimmunoassay (RIA) or enzyme-linked immunosorbent assay (ELISA). Plasma melatonin was assayed by RIA after chloroform extraction, as previously validated in European hamsters^81^. The thyroid hormones T3 and T4 were extracted with a 2:1 mixture of chloroform:methanol, purified by anion exchange chromatography, eluted with 70% acetic acid, and then measured by RIA as previously described^82^. Plasma leptin was measured using a direct multi-species RIA kit (EMD Millipore, Billerica, MA, USA), insulin using a hamster ELISA kit (Crystal Chem, Downers Grove, USA), and plasma glucose concentration using the colorimetric glucose GOD-PAP method (Biolabo, Maizy, France).

### Data and statistical analysis

Raw ddPCR data were analysed using QuantaSoft software v. 1.5.38.1118 (Biorad, Hercules, USA). Raw data were normalized against the initial quantity to obtain equivalent copy numbers for 100 ng total RNA. Statistical analyses were performed using R Program Writer software 3.3.1 (http://www.r-project.org/). Within-dataset variability was explored using two-dimensional principal component analysis (PCA) with the FactoMineR package, to identify the subset of genes showing the greatest differential expression in relation to several factors, including the organ and the hibernation phase. Data analysis was performed for each organ which was the main factors influencing expression of the genes.

Using R software, Kruskal-Wallis analysis was performed on normalized counts to determine the global effect of hibernation phases on individual gene expression in a given tissue type. Then raw *P* values from each model were adjusted for multiple testing, using Benjamini-Hochberg correction to control the false discovery rate. Genes were considered differentially expressed if the adjusted *P* values were below 0.05. Dunn’s post-hoc analysis test was used to compare further subgroups of hibernation phases.

The results of hormone measurements are presented as mean ± SD. Data were analysed using the Kruskal-Wallis test, followed by Dunn’s multiple comparison test. Statistical significance was set at *P* ≤ 0.05. Statistical analyses were performed using PRISM (GraphPad Software Inc., San Diego, CA, USA).

## Electronic supplementary material

Supplementary tables S1 & S2

## Data Availability

The datasets generated and analysed during the current study are available from the corresponding author (Jean A Boutin jean.boutin@servier.com) upon reasonable request.

## References

[1] Heldmaier G, Ortmann S, Elvert R (2004). Natural hypometabolism during hibernation and daily torpor in mammals. Respir. Physiol. Neurobiol..

[2] Geiser F, Turbill C (2009). Hibernation and daily torpor minimize mammalian extinctions. Naturwissenschaften.

[3] Grabek KR, Martin SL, Hindle AG (2015). Proteomics approaches shed new light on hibernation physiology. J. Comp. Physiol. B.

[4] Serkova NJ, Rose JC, Epperson LE, Carey HV, Martin SL (2007). Quantitative analysis of liver metabolites in three stages of the circannual hibernation cycle in 13-lined ground squirrels by NMR. Physiol. Genomics.

[5] Geiser, F. Hibernation: Endotherms. In *eLS* (ed. John Wiley & Sons, Ltd) (John Wiley & Sons, Ltd, 2011).

[6] Geiser F (2004). Metabolic Rate and Body Temperature Reduction During Hibernation and Daily Torpor. Annu. Rev. Physiol..

[7] Florant GL (2004). Fat-cell mass, serum leptin and adiponectin changes during weight gain and loss in yellow-bellied marmots (*Marmota flaviventris*). J. Comp. Physiol. B.

[8] Humphries MM, Thomas DW, Kramer DL (2003). The Role of Energy Availability in Mammalian Hibernation: A Cost/Benefit Approach. Physiol. Biochem. Zool..

[9] Canguilhem B, Petrovic A (1974). [Effects of photoperiod and ambient temperature on circannual rhythms of body weight and adrenal cortex activity in European hamster (*Cricetus cricetus*)]. Arch. Sci. Physiol. (Paris).

[10] Simonneaux V, Ribelayga C (2003). Generation of the melatonin endocrine message in mammals: A review of the complex regulation of melatonin synthesis by norepinephrine, peptides, and other pineal transmitters. Pharmacol. Rev..

[11] Hazlerigg D, Simonneaux V (2015). Chapter 34. Seasonal Regulation of Reproduction in Mammals. In Knobil and Neill’s Physiology of Reproduction: Two-Volume Set.

[12] Stanton TL, Daley JC, Salzman SK (1987). Prolongation of hibernation bout duration by continuous intracerebroventricular infusion of melatonin in hibernating ground squirrels. Brain Res..

[13] Pitrosky B, Delagrange P, Rettori MC, Pévet P (2003). S22153, a melatonin antagonist, dissociates different aspects of photoperiodic responses in Syrian hamsters. Behav. Brain Res..

[14] Nosjean O (2000). Identification of the melatonin-binding site MT3 as the quinone reductase 2. J. Biol. Chem..

[15] Dufourny L (2008). GPR50 is the mammalian ortholog of Mel1c: Evidence of rapid evolution in mammals. BMC Evol. Biol..

[16] Gautier C (2018). Characterization of the Mel1c melatoninergic receptor in platypus (Ornithorhynchus anatinus). PLOS ONE.

[17] Bechtold DA (2012). A role for the melatonin-related receptor GPR5050 in leptin signaling, adaptive thermogenesis, and torpor. Curr. Biol..

[18] Dardente H, Klosen P, Pévet P, Masson-Pévet M (2003). MT1 melatonin receptor mRNA expressing cells in the pars tuberalis of the European hamster: effect of photoperiod. J. Neuroendocrinol..

[19] Dardente H, Hazlerigg DG, Ebling FJ (2014). Thyroid hormone and seasonal rhythmicity. Thyroid Endocrinol..

[20] Lewis, J. E. & Ebling, F. J. P. Tanycytes As Regulators of Seasonal Cycles in Neuroendocrine Function. *Front. Neurol*. **8** (2017). 10.3389/fneur.2017.00079 PMC5344904 28344570

[21] Hanon EA (2010). Effect of photoperiod on the thyroid-stimulating hormone neuroendocrine system in the european hamster (*Cricetus cricetus*). J. Neuroendocrinol..

[22] Nishiwaki-Ohkawa T, Yoshimura T (2016). Molecular basis for regulating seasonal reproduction in vertebrates. J. Endocrinol..

[23] Hanon EA (2008). Ancestral TSH Mechanism Signals Summer in a Photoperiodic Mammal. Curr. Biol..

[24] Nakao N (2008). Thyrotrophin in the pars tuberalis triggers photoperiodic response. Nature.

[25] Ono H (2008). Involvement of thyrotropin in photoperiodic signal transduction in mice. Proc. Natl. Acad. Sci..

[26] Murphy M (2012). Effects of manipulating hypothalamic triiodothyronine concentrations on seasonal body weight and torpor cycles in siberian hamsters. Endocrinology.

[27] Bank JHH (2017). Gene expression analysis and microdialysis suggest hypothalamic triiodothyronine (T3) gates daily torpor in Djungarian hamsters (Phodopus sungorus). J. Comp. Physiol. B.

[28] Dibner C, Schibler U, Albrecht U (2010). The Mammalian Circadian Timing System: Organization and Coordination of Central and Peripheral Clocks. Annu. Rev. Physiol..

[29] Ko CH, Takahashi JS (2006). Molecular components of the mammalian circadian clock. Hum. Mol. Genet..

[30] Revel FG (2007). The circadian clock stops ticking during deep hibernation in the European hamster. Proc. Natl. Acad. Sci..

[31] Buck CL, Barnes BM (2000). Effects of ambient temperature on metabolic rate, respiratory quotient, and torpor in an arctic hibernator. Am. J. Physiol. - Regul. Integr. Comp. Physiol..

[32] Rui L (2014). Energy Metabolism in theLiver. Compr. Physiol..

[33] Lass A, Zimmermann R, Oberer M, Zechner R (2011). Lipolysis – A highly regulated multi-enzyme complex mediates the catabolism of cellular fat stores. Prog. Lipid Res..

[34] Cahill GF (2006). Fuel metabolism in starvation. Annu. Rev. Nutr..

[35] Picard F (2004). Sirt1 promotes fat mobilization in white adipocytes by repressing PPAR-γ. Nature.

[36] Rodgers JT (2005). Nutrient control of glucose homeostasis through a complex of PGC-1|[alpha]| and SIRT1. Nature.

[37] Kersten S (1999). Peroxisome proliferator–activated receptor α mediates the adaptive response to fasting. J. Clin. Invest..

[38] Badman MK (2007). Hepatic Fibroblast Growth Factor 21 Is Regulated by PPARα and Is a Key Mediator of Hepatic Lipid Metabolism in Ketotic States. Cell Metab..

[39] Inagaki T (2007). Endocrine Regulation of the Fasting Response by PPARα-Mediated Induction of Fibroblast Growth Factor 21. Cell Metab..

[40] Chutkow WA, Patwari P, Yoshioka J, Lee RT (2008). Thioredoxin-interacting Protein (Txnip) Is a Critical Regulator of Hepatic Glucose Production. J. Biol. Chem..

[41] Blouet C, Liu S-M, Jo Y-H, Chua S, Schwartz GJ (2012). TXNIP in Agrp Neurons Regulates Adiposity, Energy Expenditure, and Central Leptin Sensitivity. J. Neurosci..

[42] Hand LE (2013). Induction of the metabolic regulator txnip in fasting-induced and natural torpor. Endocrinology.

[43] Schwartz C, Hampton M, Andrews MT (2013). Seasonal and Regional Differences in Gene Expression in the Brain of a Hibernating Mammal. PLoS ONE.

[44] Cannon B, Nedergaard J (2004). Brown adipose tissue: function and physiological significance. Physiol. Rev..

[45] Hampton M, Melvin RG, Andrews MT (2013). Transcriptomic Analysis of Brown Adipose Tissue across the Physiological Extremes of Natural Hibernation. PLoS ONE.

[46] Scarpace PJ, Matheny M, Pollock BH, Tumer N (1997). Leptin increases uncoupling protein expression and energy expenditure. Am. J. Physiol. - Endocrinol. Metab..

[47] Srere HK, Wang LC, Martin SL (1992). Central role for differential gene expression in mammalian hibernation. Proc. Natl. Acad. Sci. USA.

[48] Cubuk C, Markowsky H, Herwig A (2017). Hypothalamic control systems show differential gene expression during spontaneous daily torpor and fasting-induced torpor in the Djungarian hamster (*Phodopus sungorus*). PLoS ONE.

[49] O’Hara BF (1999). Gene Expression in the Brain across the Hibernation Cycle. J. Neurosci..

[50] Weitten M, Robin J-P, Oudart H, Pévet P, Habold C (2013). Hormonal changes and energy substrate availability during the hibernation cycle of Syrian hamsters. Horm. Behav..

[51] Tahara Y, Shibata S (2018). Entrainment of the mouse circadian clock: Effects of stress, exercise, and nutrition. Free Radic. Biol. Med..

[52] Bitting L (1994). C-fos mRNA increases in the ground squirrel suprachiasmatic nucleus during arousal from hibernation. Neurosci. Lett..

[53] Bratincsák A (2007). Spatial and temporal activation of brain regions in hibernation:c-fos expression during the hibernation bout in thirteen-lined ground squirrel. J. Comp. Neurol..

[54] Ruby NF, Zucker I (1992). Daily torpor in the absence of the suprachiasmatic nucleus in Siberian hamsters. Am. J. Physiol. - Regul. Integr. Comp. Physiol..

[55] Ruby NF, Dark J, Heller HC, Zucker I (1996). Ablation of suprachiasmatic nucleus alters timing of hibernation in ground squirrels. Proc. Natl. Acad. Sci. USA.

[56] Florant GL, Rivera ML, Lawrence AK, Tamarkin L (1984). Plasma melatonin concentrations in hibernating marmots: absence of a plasma melatonin rhythm. Am. J. Physiol. - Regul. Integr. Comp. Physiol..

[57] Darrow JM, Tamarkin L, Duncan MJ, Goldman BD (1986). Pineal melatonin rhythms in female Turkish hamsters: effects of photoperiod and hibernation. Biol. Reprod..

[58] Vanĕcek J, Janský L, Illnerová H, Hoffmann K (1985). Arrest of the circadian pacemaker driving the pineal melatonin rhythm in hibernating golden hamsters, Mesocricetus auratus. Comp. Biochem. Physiol. A.

[59] Gauer F, Masson-Pévet M, Saboureau M, George D, Pévet P (1993). Differential seasonal regulation of melatonin receptor density in the pars tuberalis and the suprachiasmatic nuclei: a study in the hedgehog (*Erinaceus europaeus, L*.). J. Neuroendocrinol..

[60] Stanton TL, Siuciak JA, Dubocovich ML, Krause DN (1991). The area of 2-[125I]iodomelatonin binding in the pars tuberalis of the ground squirrel is decreased during hibernation. Brain Res..

[61] Schwartz C, Ballinger MA, Andrews MT (2015). Melatonin receptor signaling contributes to neuroprotection upon arousal from torpor in thirteen-lined ground squirrels. Am. J. Physiol. - Regul. Integr. Comp. Physiol..

[62] Luan Y (2018). Integrated transcriptomic and metabolomic analysis reveals adaptive changes of hibernating retinas. J. Cell. Physiol..

[63] Merriman DK, Sajdak BS, Li W, Jones BW (2016). Seasonal and post-trauma remodeling in cone-dominant ground squirrel retina. Exp. Eye Res..

[64] Remé CE, Young RW (1977). The effects of hibernation on cone visual cells in the ground squirrel. Invest. Ophthalmol. Vis. Sci..

[65] Cipolla‐Neto J, Amaral FG, Afeche SC, Tan DX, Reiter RJ (2014). Melatonin, energy metabolism, and obesity: a review. J. Pineal Res..

[66] Heldmaier G, Steinlechner S, Rafael J, Vsiansky P (1981). Photoperiodic control and effects of melatonin on nonshivering thermogenesis and brown adipose tissue. Science.

[67] McMillan AC, White MD (2015). Induction of thermogenesis in brown and beige adipose tissues: molecular markers, mild cold exposure and novel therapies. Curr. Opin. Endocrinol. Diabetes Obes..

[68] Tan D-X, Manchester LC, Fuentes-Broto L, Paredes SD, Reiter RJ (2011). Significance and application of melatonin in the regulation of brown adipose tissue metabolism: relation to human obesity. Obes. Rev..

[69] Ivanova EA (2008). Altered metabolism in the melatonin-related receptor (GPR50) knockout mouse. Am. J. Physiol. - Endocrinol. Metab..

[70] Barrett P (2007). Hypothalamic thyroid hormone catabolism acts as a gatekeeper for the seasonal control of body weight and reproduction. Endocrinology.

[71] Klosen P, Sébert M-E, Rasri K, Laran-Chich M-P, Simonneaux V (2013). TSH restores a summer phenotype in photoinhibited mammals via the RF-amides RFRP3 and kisspeptin. FASEB J..

[72] Bank JHH, Kemmling J, Rijntjes E, Wirth EK, Herwig A (2015). Thyroid hormone status affects expression of daily torpor and gene transcription in Djungarian hamsters (Phodopus sungorus). Horm. Behav..

[73] Puigserver P, Spiegelman BM (2003). Peroxisome Proliferator-Activated Receptor-γ Coactivator 1α (PGC-1α): Transcriptional Coactivator and Metabolic Regulator. Endocr. Rev..

[74] Honma S (2002). *Dec1* and *Dec2* are regulators of the mammalian molecular clock. Nature.

[75] Jastroch, M. *et al*. Seasonal Control of Mammalian Energy Balance: Recent Advances in the Understanding of Daily Torpor and Hibernation. *J. Neuroendocrinol*. **28** (2016). 10.1111/jne.12437 27755687

[76] Hindle AG, Grabek KR, Epperson LE, Karimpour-Fard A, Martin SL (2014). Metabolic changes associated with the long winter fast dominate the liver proteome in 13-lined ground squirrels. Physiol. Genomics.

[77] Bouma HR (2012). Induction of torpor: mimicking natural metabolic suppression for biomedical applications. J. Cell. Physiol..

[78] Dave KR, Christian SL, Perez-Pinzon MA, Drew KL (2012). Neuroprotection: Lessons from hibernators. Comp. Biochem. Physiol. B Biochem. Mol. Biol..

[79] Ivakine EA, Cohn RD (2014). Maintaining skeletal muscle mass: lessons learned from hibernation. Exp. Physiol..

[80] Canguilhem B, Marx C (1973). Regulation of the body weight of the European Hamster during the annual cycle. Pflugers Arch..

[81] Garidou M-L, Vivien-Roels B, Pévet P, Miguez J, Simonneaux V (2003). Mechanisms regulating the marked seasonal variation in melatonin synthesis in the European hamster pineal gland. Am. J. Physiol. - Regul. Integr. Comp. Physiol..

[82] Reyns GE, Venken K, Morreale de Escobar G, Kühn ER, Darras VM (2003). Dynamics and regulation of intracellular thyroid hormone concentrations in embryonic chicken liver, kidney, brain, and blood. Gen. Comp. Endocrinol..

