# Supplementary material for: Gene expression profiling during hibernation in the European hamster
Source: Sci Rep. 2018 Sep 3;8:13167. doi: 10.1038/s41598-018-31506-2 (PMC6120936; doi:10.1038/s41598-018-31506-2)
Supplement: Supplementary file 1 — Supplementary tables S1 & S2 [file 41598_2018_31506_MOESM1_ESM.pdf]

Gene expression profiling during hibernation in the European hamster.

Célia Gautier, Béatrice Bothorel, Dominique Ciocca, Damien Valour, Albane Gaudeau, Clémence Dupré, Giulia Lizzo, Chantal Brasseur, Isabelle Riest-Fery, Jean-Philippe Stephan, Olivier Nosjean, Jean A. Boutin, Sophie-Pénélope Guénin, Valérie Simonneaux

**Table S1:** Median, first quartile (25 %), third quartile (75 %), and interquartile (Q75%-Q25%) of absolute RNA copies of each gene in each organ at each hibernation state (N=Normothermia, T=Torpor, A= Arousal).

| | | **Per1** | | | | **Bmal1** | | | | **Rev-Erbα** | | | |
|---|---|---|---|---|---|---|---|---|---|---|---|---|---|
| | | **Median** | **Quartile (25%)** | **Quartile (75%)** | **Interquartile (Q75%-Q25%)** | **Median** | **Quartile (25%)** | **Quartile (75%)** | **Interquartile (Q75%-Q25%)** | **Median** | **Quartile (25%)** | **Quartile (75%)** | **Interquartile (Q75%-Q25%)** |
| **Cerebral Cortex** | **N** | 41100 | 27200 | 54150 | 26950 | 4960 | 2540 | 5210 | 2670 | 58000 | 43450 | 76600 | 33150 |
| | **T** | 63300 | 55000 | 97700 | 42700 | 4040 | 3270 | 5620 | 2350 | 47100 | 41500 | 85000 | 43500 |
| | **A** | 91300 | 77600 | 151550 | 73950 | 3860 | 3650 | 5250 | 1600 | 52500 | 40050 | 63450 | 23400 |
| **Hypothalamus** | **N** | 43600 | 30450 | 60550 | 30100 | 8040 | 6300 | 8920 | 2620 | 121800 | 90000 | 146600 | 56600 |
| | **T** | 66400 | 28400 | 88100 | 59700 | 3640 | 1360 | 4510 | 3150 | 53400 | 24400 | 74250 | 49850 |
| | **A** | 110700 | 102000 | 113200 | 11200 | 4080 | 3600 | 6620 | 3020 | 58800 | 46850 | 68650 | 21800 |
| **Pituitary gland** | **N** | 140200 | 107800 | 174400 | 66600 | 5720 | 4560 | 6140 | 1580 | 202000 | 169000 | 239000 | 70000 |
| | **T** | 488000 | 447000 | 536500 | 89500 | 6980 | 5840 | 7780 | 1940 | 279000 | 244500 | 309000 | 64500 |
| | **A** | 392000 | 363000 | 490000 | 127000 | 18080 | 7980 | 22680 | 14700 | 167000 | 164500 | 176000 | 11500 |
| **Retina** | **N** | 180000 | 155100 | 253000 | 97900 | 63000 | 58600 | 67800 | 9200 | 200000 | 185000 | 258000 | 73000 |
| | **T** | 269000 | 230500 | 303500 | 73000 | 58800 | 41230 | 66800 | 25570 | 173600 | 141600 | 194000 | 52400 |
| | **A** | 336000 | 298000 | 397000 | 99000 | 93800 | 85700 | 108400 | 22700 | 192000 | 172250 | 204500 | 32250 |
| **Liver** | **N** | 13900 | 10000 | 20700 | 10700 | 1440 | 780 | 2610 | 1830 | 38200 | 29400 | 48650 | 19250 |
| | **T** | 38100 | 28550 | 57400 | 28850 | 2180 | 1520 | 3520 | 2000 | 38800 | 23650 | 58400 | 34750 |
| | **A** | 31300 | 27350 | 44450 | 17100 | 1640 | 1290 | 1950 | 660 | 22000 | 16650 | 39600 | 22950 |
| **BAT** | **N** | 28200 | 22500 | 31200 | 8700 | 1080 | 740 | 1480 | 740 | 46600 | 46400 | 60000 | 13600 |
| | **T** | 87800 | 52500 | 96750 | 44250 | 1380 | 1070 | 1620 | 550 | 69500 | 55950 | 77700 | 21750 |
| | **A** | 75400 | 53800 | 111950 | 58150 | 1140 | 870 | 2390 | 1520 | 52200 | 40900 | 61600 | 20700 |
| **Heart** | **N** | 27000 | 22500 | 32300 | 9800 | 1600 | 1220 | 1920 | 700 | 55000 | 48700 | 60700 | 12000 |
| | **T** | 100400 | 83750 | 134850 | 51100 | 1720 | 960 | 2760 | 1800 | 59500 | 50600 | 66200 | 15600 |
| | **A** | 99700 | 83100 | 144900 | 61800 | 1820 | 1110 | 2240 | 1130 | 32300 | 26150 | 39350 | 13200 |
| **Adrenal glands** | **N** | 99200 | 59000 | 126050 | 67050 | 3660 | 2440 | 4520 | 2080 | 94200 | 74600 | 110050 | 35450 |
| | **T** | 275000 | 226500 | 347000 | 120500 | 7700 | 6550 | 8250 | 1700 | 94900 | 76400 | 112350 | 35950 |
| | **A** | 313000 | 138600 | 399500 | 260900 | 7380 | 3400 | 10310 | 6910 | 97400 | 48800 | 118900 | 70100 |

**Table S1(Cont'd) :** Median, first quartile (25 %), third quartile (75 %), and interquartile (Q75%-Q25%) of absolute RNA copies of each gene in each organ at each hibernation state

| | | Clock | | | | Per2 | | | | MT1 | | | |
|---|---|---|---|---|---|---|---|---|---|---|---|---|---|
| | | **Median** | **Quartile (25%)** | **Quartile (75%)** | **Interquartile (Q75%-Q25%)** | **Median** | **Quartile (25%)** | **Quartile (75%)** | **Interquartile (Q75%-Q25%)** | **Median** | **Quartile (25%)** | **Quartile (75%)** | **Interquartile (Q75%-Q25%)** |
| **Cerebral Cortex** | **N** | 54720 | 48900 | 69340 | 20440 | 14010 | 11895 | 21350 | 9455 | 91 | 76 | 135 | 60 |
| | **T** | 41020 | 37030 | 47350 | 10320 | 17420 | 16270 | 25900 | 9630 | 111 | 86 | 128 | 42 |
| | **A** | 51180 | 44530 | 72020 | 27490 | 40300 | 32650 | 47350 | 14700 | 105 | 79 | 129 | 50 |
| **Hypothalamus** | **N** | 102600 | 67820 | 119600 | 51780 | 25000 | 14685 | 31750 | 17065 | 349 | 283 | 406 | 123 |
| | **T** | 41280 | 31600 | 50160 | 18560 | 18100 | 11520 | 22500 | 10980 | 227 | 156 | 259 | 103 |
| | **A** | 64160 | 49950 | 77420 | 27470 | 48600 | 41850 | 60400 | 18550 | 251 | 212 | 399 | 187 |
| **Pituitary gland** | **N** | 168000 | 159400 | 169400 | 10000 | 45400 | 36100 | 49300 | 13200 | 3093 | 2167 | 3313 | 1147 |
| | **T** | 144400 | 126400 | 155000 | 28600 | 109700 | 94900 | 126100 | 31200 | 2107 | 1432 | 2250 | 818 |
| | **A** | 164800 | 163400 | 174600 | 11200 | 143400 | 95400 | 183400 | 88000 | 2280 | 2140 | 3133 | 993 |
| **Retina** | **N** | 217600 | 190100 | 264300 | 74200 | 49000 | 45300 | 54800 | 9500 | 507 | 324 | 1740 | 1416 |
| | **T** | 172000 | 160900 | 177000 | 16100 | 55700 | 43350 | 60750 | 17400 | 425 | 384 | 658 | 274 |
| | **A** | 220000 | 213800 | 238100 | 24300 | 131700 | 103200 | 155700 | 52500 | 405 | 330 | 460 | 130 |
| **Liver** | **N** | 23380 | 18600 | 29500 | 10900 | 7740 | 6675 | 10355 | 3680 | 67 | 28 | 80 | 52 |
| | **T** | 21480 | 17340 | 26530 | 9190 | 24200 | 7350 | 33900 | 26550 | 33 | 28 | 50 | 22 |
| | **A** | 23580 | 18720 | 25090 | 6370 | 29500 | 25450 | 44800 | 19350 | 37 | 32 | 53 | 22 |
| **BAT** | **N** | 56480 | 46290 | 65140 | 18850 | 17500 | 15725 | 22050 | 6325 | 69 | 34 | 148 | 114 |
| | **T** | 42900 | 33680 | 46760 | 13080 | 22200 | 19800 | 23700 | 3900 | 39 | 28 | 68 | 40 |
| | **A** | 60300 | 47780 | 71190 | 23410 | 56500 | 47500 | 59200 | 11700 | 27 | 12 | 56 | 44 |
| **Heart** | **N** | 58740 | 52360 | 67010 | 14650 | 6150 | 5380 | 8105 | 2725 | 150 | 113 | 225 | 113 |
| | **T** | 51540 | 37500 | 57710 | 20210 | 12570 | 9070 | 15275 | 6205 | 188 | 130 | 195 | 65 |
| | **A** | 66500 | 54280 | 68240 | 13960 | 46700 | 36950 | 49900 | 12950 | 149 | 123 | 176 | 53 |
| **Adrenal glands** | **N** | 76960 | 48080 | 93010 | 44930 | 26100 | 19400 | 33100 | 13700 | 263 | 178 | 354 | 176 |
| | **T** | 62840 | 53460 | 74750 | 21290 | 47100 | 35650 | 50400 | 14750 | 112 | 100 | 123 | 23 |
| | **A** | 90740 | 54410 | 96500 | 42090 | 103700 | 69150 | 138850 | 69700 | 147 | 104 | 264 | 159 |

**Table S1 (cont'd):** Median, first quartile (25 %), third quartile (75 %), and interquartile (Q75%-Q25%) of absolute RNA copies of each gene in each organ at each hibernation state

| | | MT2 | | | | GPR50 | | | | QR2 | | | |
|---|---|---|---|---|---|---|---|---|---|---|---|---|---|
| | | Median | Quartile (25%) | Quartile (75%) | Interquartile (Q75%-Q25%) | Median | Quartile (25%) | Quartile (75%) | Interquartile (Q75%-Q25%) | Median | Quartile (25%) | Quartile (75%) | Interquartile (Q75%-Q25%) |
| Cerebral Cortex | N | 646 | 561 | 954 | 393 | 13 | 9 | 16 | 7 | 8460 | 7060 | 9710 | 2650 |
| | T | 537 | 486 | 681 | 196 | 27 | 19 | 30 | 11 | 8840 | 7170 | 10190 | 3020 |
| | A | 1005 | 698 | 1245 | 547 | 39 | 27 | 45 | 19 | 6760 | 5590 | 8750 | 3160 |
| Hypothalamus | N | 688 | 548 | 880 | 332 | 129 | 79 | 165 | 85 | 20900 | 14610 | 22770 | 8160 |
| | T | 542 | 354 | 638 | 284 | 80 | 23 | 85 | 63 | 12160 | 9480 | 15480 | 6000 |
| | A | 1099 | 880 | 1522 | 642 | 115 | 86 | 156 | 69 | 8700 | 7540 | 9730 | 2190 |
| Pituitary gland | N | 796 | 678 | 1224 | 546 | 485 | 385 | 645 | 260 | 29200 | 27960 | 30460 | 2500 |
| | T | 725 | 606 | 942 | 336 | 295 | 209 | 360 | 150 | 25200 | 23720 | 27000 | 3280 |
| | A | 1128 | 818 | 1455 | 637 | 527 | 411 | 742 | 331 | 22080 | 19700 | 24400 | 4700 |
| Retina | N | 751 | 460 | 1605 | 1145 | 202 | 111 | 515 | 404 | 22640 | 16800 | 26470 | 9670 |
| | T | 723 | 657 | 965 | 308 | 262 | 230 | 310 | 80 | 20900 | 19020 | 24620 | 5600 |
| | A | 1731 | 1543 | 2050 | 508 | 351 | 325 | 393 | 69 | 13820 | 10660 | 15860 | 5200 |
| Liver | N | 239 | 221 | 374 | 153 | 21 | 17 | 28 | 12 | 59800 | 49800 | 72300 | 22500 |
| | T | 67 | 52 | 137 | 86 | 10 | 5 | 15 | 10 | 62200 | 55700 | 82900 | 27200 |
| | A | 88 | 65 | 102 | 38 | 11 | 9 | 26 | 18 | 42200 | 39800 | 50100 | 10300 |
| BAT | N | 295 | 263 | 352 | 89 | 25 | 11 | 42 | 31 | 34400 | 30800 | 41000 | 10200 |
| | T | 127 | 94 | 136 | 42 | 12 | 9 | 26 | 17 | 38600 | 34480 | 43200 | 8720 |
| | A | 109 | 82 | 127 | 46 | 17 | 11 | 36 | 26 | 28540 | 23210 | 31180 | 7970 |
| Heart | N | 337 | 279 | 377 | 98 | 25 | 19 | 36 | 16 | 21000 | 16720 | 27830 | 11110 |
| | T | 213 | 168 | 243 | 75 | 27 | 22 | 32 | 10 | 24960 | 18310 | 28930 | 10620 |
| | A | 225 | 206 | 270 | 64 | 24 | 23 | 29 | 6 | 22460 | 17560 | 24060 | 6500 |
| Adrenal glands | N | 290 | 252 | 408 | 157 | 62 | 36 | 93 | 57 | 34800 | 24370 | 43600 | 19230 |
| | T | 216 | 173 | 236 | 63 | 47 | 43 | 54 | 10 | 39600 | 32920 | 43500 | 10580 |
| | A | 240 | 205 | 341 | 136 | 67 | 52 | 92 | 40 | 34600 | 24910 | 40200 | 15290 |

**Table S1(cont'd):** Median, first quartile (25 %), third quartile (75 %), and interquartile (Q75%-Q25%) of absolute RNA copies of each gene in each organ at each hibernation state

| | | SIRT1 | | | | TXNIP | | | | PPARα | | | |
|---|---|---|---|---|---|---|---|---|---|---|---|---|---|
| | | Median | Quartile (25%) | Quartile (75%) | Interquartile (Q75%-Q25%) | Median | Quartile (25%) | Quartile (75%) | Interquartile (Q75%-Q25%) | Median | Quartile (25%) | Quartile (75%) | Interquartile (Q75%-Q25%) |
| Cerebral Cortex | N | 27800 | 19970 | 31800 | 11830 | 41700 | 31150 | 60350 | 29200 | 5600 | 4950 | 6750 | 1800 |
| | T | 23200 | 20630 | 26460 | 5830 | 50600 | 44100 | 73550 | 29450 | 3400 | 2350 | 4700 | 2350 |
| | A | 30200 | 25760 | 40400 | 14640 | 32900 | 26400 | 39350 | 12950 | 6100 | 5000 | 7800 | 2800 |
| Hypothalamus | N | 64000 | 45900 | 68700 | 22800 | 96700 | 71650 | 143900 | 72250 | 9800 | 7350 | 13800 | 6450 |
| | T | 31100 | 22080 | 38760 | 16680 | 66000 | 29200 | 103700 | 74500 | 2600 | 320 | 4150 | 3830 |
| | A | 40400 | 36040 | 47900 | 11860 | 50200 | 32450 | 59150 | 26700 | 7100 | 4900 | 9250 | 4350 |
| Pituitary gland | N | 80800 | 73000 | 90000 | 17000 | 516000 | 439000 | 743000 | 304000 | 17800 | 15500 | 21100 | 5600 |
| | T | 84800 | 82800 | 94500 | 11700 | 754000 | 642500 | 932000 | 289500 | 16500 | 14800 | 17650 | 2850 |
| | A | 92400 | 91200 | 100400 | 9200 | 420000 | 375000 | 465000 | 90000 | 21800 | 17900 | 24900 | 7000 |
| Retina | N | 160600 | 148200 | 175100 | 26900 | 25700 | 24250 | 28400 | 4150 | 66000 | 65200 | 73350 | 8150 |
| | T | 135600 | 118000 | 151300 | 33300 | 36500 | 31150 | 43600 | 12450 | 24200 | 20450 | 25600 | 5150 |
| | A | 199200 | 107400 | 211400 | 104000 | 28400 | 21350 | 35200 | 13850 | 39300 | 31100 | 66450 | 35350 |
| Liver | N | 12800 | 7720 | 14700 | 6980 | 29800 | 22850 | 50350 | 27500 | 104300 | 94600 | 119500 | 24900 |
| | T | 19340 | 15890 | 22440 | 6550 | 50000 | 35050 | 89250 | 54200 | 91300 | 49900 | 131050 | 81150 |
| | A | 16800 | 15490 | 19870 | 4380 | 42900 | 19050 | 119550 | 100500 | 69700 | 59300 | 88550 | 29250 |
| BAT | N | 35000 | 27480 | 45400 | 17920 | 590000 | 501500 | 719000 | 217500 | 383000 | 336500 | 449000 | 112500 |
| | T | 30380 | 20100 | 35110 | 15010 | 550000 | 385500 | 642000 | 256500 | 273000 | 223700 | 290000 | 66300 |
| | A | 51200 | 38200 | 63900 | 25700 | 513000 | 374000 | 603000 | 229000 | 185000 | 141350 | 209000 | 67650 |
| Heart | N | 29500 | 26200 | 38400 | 12200 | 366000 | 219000 | 457500 | 238500 | 28500 | 23400 | 34400 | 11000 |
| | T | 30760 | 21660 | 33900 | 12240 | 281000 | 175000 | 416000 | 241000 | 20400 | 14800 | 22750 | 7950 |
| | A | 34400 | 31710 | 37900 | 6190 | 147700 | 102900 | 187500 | 84600 | 22800 | 19600 | 31500 | 11900 |
| Adrenal glands | N | 43200 | 30190 | 51400 | 21210 | 206000 | 145400 | 264500 | 119100 | 12200 | 8900 | 15400 | 6500 |
| | T | 40600 | 36700 | 46800 | 10100 | 146100 | 128650 | 222500 | 93850 | 9800 | 8600 | 10650 | 2050 |
| | A | 57400 | 36200 | 64600 | 28400 | 201000 | 169400 | 245000 | 75600 | 14900 | 12400 | 19950 | 7550 |

**Table S1(cont'd):** Median, first quartile (25 %), third quartile (75 %), and interquartile (Q75%-Q25%) of absolute RNA copies of each gene in each organ at each hibernation state

| | | PGC1α | | | | FGF21 | | | | ObR | | | |
|---|---|---|---|---|---|---|---|---|---|---|---|---|---|
| | | **Median** | **Quartile (25%)** | **Quartile (75%)** | **Interquartile (Q75%-Q25%)** | **Median** | **Quartile (25%)** | **Quartile (75%)** | **Interquartile (Q75%-Q25%)** | **Median** | **Quartile (25%)** | **Quartile (75%)** | **Interquartile (Q75%-Q25%)** |
| **Cerebral Cortex** | **N** | 26200 | 18550 | 36900 | 18350 | 198 | 156 | 284 | 128 | 1159 | 972 | 1583 | 611 |
| | **T** | 13000 | 10850 | 17650 | 6800 | 189 | 145 | 269 | 125 | 1291 | 839 | 1605 | 767 |
| | **A** | 20100 | 17300 | 29500 | 12200 | 264 | 219 | 410 | 191 | 1287 | 1097 | 1451 | 354 |
| **Hypothalamus** | **N** | 46900 | 42200 | 54550 | 12350 | 278 | 235 | 337 | 103 | 2160 | 1716 | 2675 | 959 |
| | **T** | 13800 | 7400 | 19500 | 12100 | 179 | 116 | 208 | 92 | 2250 | 1376 | 2595 | 1219 |
| | **A** | 26300 | 16650 | 29850 | 13200 | 330 | 282 | 368 | 86 | 1363 | 1066 | 1810 | 744 |
| **Pituitary gland** | **N** | 5400 | 4300 | 6900 | 2600 | 630 | 522 | 1235 | 713 | 5200 | 4450 | 6360 | 1910 |
| | **T** | 5500 | 3600 | 6550 | 2950 | 1371 | 1144 | 1494 | 350 | 3640 | 3445 | 3840 | 395 |
| | **A** | 5600 | 5300 | 6800 | 1500 | 1900 | 1560 | 2180 | 620 | 3680 | 2950 | 3940 | 990 |
| **Retina** | **N** | 58000 | 51150 | 82800 | 31650 | 708 | 551 | 986 | 436 | 3500 | 2335 | 4810 | 2475 |
| | **T** | 36200 | 32550 | 49250 | 16700 | 1544 | 1169 | 1805 | 636 | 2550 | 2055 | 3130 | 1075 |
| | **A** | 53000 | 45750 | 60550 | 14800 | 2800 | 2310 | 3700 | 1390 | 2520 | 2435 | 2745 | 310 |
| **Liver** | **N** | 1410 | 780 | 2100 | 1320 | 5470 | 3230 | 6975 | 3745 | 10070 | 7700 | 11420 | 3720 |
| | **T** | 2500 | 1090 | 3250 | 2160 | 1043 | 804 | 1527 | 723 | 27240 | 23955 | 31305 | 7350 |
| | **A** | 3400 | 1750 | 4250 | 2500 | 11840 | 1715 | 16295 | 14581 | 22240 | 18935 | 36290 | 17355 |
| **BAT** | **N** | 57600 | 50700 | 63900 | 13200 | 211 | 180 | 289 | 109 | 449 | 315 | 558 | 244 |
| | **T** | 46800 | 32600 | 52300 | 19700 | 127 | 111 | 159 | 48 | 417 | 374 | 538 | 164 |
| | **A** | 86600 | 51300 | 154500 | 103200 | 164 | 136 | 199 | 63 | 271 | 220 | 322 | 102 |
| **Heart** | **N** | 49900 | 37950 | 59850 | 21900 | 4260 | 3630 | 5550 | 1920 | 1421 | 1323 | 1625 | 303 |
| | **T** | 40300 | 33700 | 54050 | 20350 | 4770 | 2720 | 5805 | 3085 | 1393 | 1006 | 1700 | 694 |
| | **A** | 36500 | 29550 | 42150 | 12600 | 3830 | 3230 | 5195 | 1965 | 979 | 782 | 1124 | 343 |
| **Adrenal glands** | **N** | 1590 | 1035 | 1700 | 665 | 467 | 352 | 640 | 288 | 1480 | 1136 | 1745 | 610 |
| | **T** | 930 | 640 | 1460 | 820 | 217 | 203 | 366 | 163 | 930 | 783 | 1191 | 409 |
| | **A** | 1710 | 1500 | 2000 | 500 | 1030 | 534 | 1505 | 972 | 757 | 609 | 993 | 384 |

**Table S1(end):** Median, first quartile (25 %), third quartile (75 %), and interquartile (Q75%-Q25%) of absolute RNA copies of each gene in each organ at each hibernation state

| | | Dio2 | | | | Dio3 | | | | UCP1 | | | |
|---|---|---|---|---|---|---|---|---|---|---|---|---|---|
| | | **Median** | **Quartile (25%)** | **Quartile (75%)** | **Interquartile (Q75%-Q25%)** | **Median** | **Quartile (25%)** | **Quartile (75%)** | **Interquartile (Q75%-Q25%)** | **Median** | **Quartile (25%)** | **Quartile (75%)** | **Interquartile (Q75%-Q25%)** |
| **Cerebral Cortex** | **N** | 2620 | 2180 | 3175 | 995 | 790 | 526 | 991 | 465 | | | | |
| | **T** | 1760 | 1625 | 2175 | 550 | 760 | 258 | 1088 | 830 | | | | |
| | **A** | 1440 | 1110 | 1755 | 645 | 684 | 627 | 1110 | 484 | | | | |
| **Hypothalamus** | **N** | 1850 | 1615 | 2430 | 815 | 461 | 289 | 693 | 404 | | | | |
| | **T** | 920 | 660 | 1045 | 385 | 410 | 132 | 543 | 411 | | | | |
| | **A** | 1130 | 695 | 1495 | 800 | 463 | 214 | 873 | 659 | | | | |
| **Pituitary gland** | **N** | 4220 | 3520 | 4830 | 1310 | 310 | 244 | 525 | 281 | | | | |
| | **T** | 1680 | 1365 | 2100 | 735 | 218 | 187 | 286 | 100 | | | | |
| | **A** | 2340 | 1610 | 3030 | 1420 | 320 | 259 | 657 | 398 | | | | |
| **Retina** | **N** | 2950 | 1640 | 4395 | 2755 | 2320 | 1865 | 2650 | 786 | | | | |
| | **T** | 1560 | 1415 | 1805 | 390 | 1550 | 1460 | 1925 | 465 | | | | |
| | **A** | 1880 | 1475 | 2730 | 1255 | 2000 | 1875 | 2245 | 370 | | | | |
| **Liver** | **N** | 164 | 92 | 270 | 178 | 39 | 26 | 43 | 17 | | | | |
| | **T** | 60 | 30 | 105 | 76 | 35 | 28 | 62 | 34 | | | | |
| | **A** | 88 | 51 | 111 | 60 | 39 | 36 | 46 | 10 | | | | |
| **BAT** | **N** | 5780 | 4375 | 6375 | 2000 | 101 | 66 | 129 | 64 | 2970000 | 1680000 | 3825000 | 2145000 |
| | **T** | 1760 | 1605 | 2255 | 650 | 83 | 74 | 87 | 13 | 3270000 | 2405000 | 3790000 | 1385000 |
| | **A** | 1330 | 1180 | 1650 | 470 | 236 | 174 | 344 | 170 | 2370000 | 1860000 | 3375000 | 1515000 |
| **Heart** | **N** | 670 | 535 | 795 | 260 | 61 | 46 | 101 | 56 | | | | |
| | **T** | 530 | 405 | 630 | 225 | 114 | 82 | 145 | 63 | | | | |
| | **A** | 250 | 160 | 325 | 165 | 129 | 112 | 147 | 35 | | | | |
| **Adrenal glands** | **N** | 280 | 180 | 430 | 250 | 81 | 54 | 126 | 72 | | | | |
| | **T** | 44 | 12 | 138 | 126 | 80 | 51 | 105 | 54 | | | | |
| | **A** | 44 | 12 | 160 | 148 | 160 | 92 | 204 | 112 | | | | |

Gene expression profiling during hibernation in the European hamster

Célia Gautier[1,2], Béatrice Bothorel[2], Dominique Ciocca[2], Damien Valour[3], Albane Gaudeau[1], Clémence Dupré[1], Giulia Lizzo[1], Chantal Brasseur[1], Isabelle Riest-Fery[1], Jean-Philippe Stephan[1], Olivier Nosjean[1,4], Jean A. Boutin[1,4]*, Sophie-Pénélope Guénin[1,+], Valérie Simonneaux[2,+]*

**Supplementary Table S2**: Plasma hormone levels (means± SD) of European hamster during the hibernation cycle (n=5 - 8).

| | **Normothermia** | **Torpor** | **Arousal** |
|---|---|---|---|
| **T3** (pmol/ml) | 1,20 ± 0,18 | 1,08 ± 0,20 | 1,02 ± 0,12 |
| **T4** (pmol/ml) | 28,19 ± 4,85 | 19,31 ± 2,68 | 17,69 ± 3,54 |
| **Glucose** (mmol/ml) | 5,70 ± 2,69 | 3,42 ± 0,78 | 4,32 ± 1,74 |
| **Insulin** (ng/ml) | 1,70 ± 0,99 | 0,80 ± 0,41 | 1,76 ± 1,38 |
| **Leptin** (ng/ml) | 28,30 ± 8,05 | 14,28 ± 4,99 | 27,69 ± 7,06 |
| **Melatonin** (pg/ml) | 23,16 ± 27,78 | 18,83 ± 10,22 | 37,67 ± 38,71 |
